# *Staphylococcus aureus* pore-forming toxins differentially shape disease severity in experimental endophthalmitis

**DOI:** 10.1128/iai.00168-26

**Published:** 2026-06-22

**Authors:** Luis Longoria-Gonzalez, Van Le, Aaron C. Parrot, Phillip S. Coburn, Roger Astley, Michelle C. Callegan

**Affiliations:** 1 Department of Microbiology and Immunology, University of Oklahoma Health Campus 6186, Oklahoma City, Oklahoma, USA; 2 Department of Ophthalmology, University of Oklahoma Health Campus 6186, Oklahoma City, Oklahoma, USA; 3 Dean McGee Eye Institute 44201, Oklahoma City, Oklahoma, USA; University of Illinois Chicago, Chicago, Illinois, USA

**Keywords:** *Staphylococcus aureus*, endophthalmitis, leukocidin, alpha toxin, inflammation

## Abstract

Ocular infections caused by *Staphylococcus aureus* result in poor visual outcomes due to the expression of numerous virulence factors during infection. We hypothesized that pore-forming toxins (PFTs) contribute to the pathogenesis of *S. aureus* endophthalmitis, a severe intraocular infection that can result in blindness. We analyzed infection outcomes using wild-type (LAC), PFT-null (LAC ΔΔΔΔΔ), leukocidin-null (LAC Δ*leukocidin*), or alpha toxin-deficient (LAC Δ*hla*) mutants. These strains exhibited no differences in growth in brain heart infusion (BHI) and explanted rabbit vitreous. The expression of PFT genes was detected in both environments, but transcript levels were less in vitreous relative to that of BHI. Experimental endophthalmitis was induced in C57BL6/J mice by intravitreal injections of 5,000 colony-forming units (CFU) of *S. aureus* LAC or one of its three mutants. To quantify infection severity, eyes were analyzed for retinal function, intraocular CFU, inflammation via myeloperoxidase (MPO), cytokines and chemokines, neutrophil infiltration, alpha toxin production, and gross pathology via histology and slit-lamp imaging. Infections with LAC ΔΔΔΔΔ or LAC Δ*hla* resulted in improved retinal function, reduced intraocular CFUs, decreased ocular damage, and a trend toward decreased inflammation compared to infections with LAC. In contrast, infections with LAC Δ*leukocidin* progressed similarly as infections with LAC. Increasing concentrations of alpha toxin were detected over time in the eyes infected with LAC and LAC Δ*leukocidin*; 40%–60% of infiltrating neutrophils were ADAM10^+^, identifying these cells as potential targets for alpha toxin. Collectively, these results suggest that alpha toxin may be the main PFT driving the severity of *S. aureus* endophthalmitis.

## INTRODUCTION

As a genus, staphylococci are primary causative agents of ocular infections including endophthalmitis as these bacteria are part of the ocular and periocular microbiota (1, 2). Endophthalmitis is a severe intraocular infection that may damage sensitive and non-regenerating intraocular tissue, which can result in permanent vision loss, and in worst-case scenarios, loss of the infected eye (3). Endophthalmitis most commonly occurs due to complications from intraocular procedures, such as cataract surgery or intravitreal injections (post-operative endophthalmitis), but may also be a consequence of a penetrating eye injury (post-traumatic endophthalmitis) or hematogenous spread from a systemic infection (endogenous endophthalmitis) (4–7).

At the time of diagnosis, the causative microorganism is usually unknown. As a result, clinicians empirically treat endophthalmitis with intravitreal injections of vancomycin and ceftazidime to target both gram-positive and gram-negative bacteria (8). Coagulase-negative staphylococci (CoNS) are among the most common organisms isolated from ocular infections, accounting for about 70% of organisms isolated from post-cataract surgery endophthalmitis. *Staphylococcus aureus* (*S. aureus*) accounts for only 10% of organisms isolated from cataract surgery-associated endophthalmitis cases (3). *S. aureus* is also isolated in 25% of endogenous endophthalmitis cases, which can result in bilateral blindness (9). Patients with endophthalmitis develop acute ocular inflammation and redness, eye pain, and rapidly decreasing vision (3, 10). Ocular infections with CoNS typically resolve well with minimal impact on vision. In contrast, *S. aureus* ocular infections can lead to severe intraocular inflammation and retinal damage, resulting in poor visual outcomes. In these infections, final visual acuities have been reported to be 20/100 (i.e., moderate visual impairment) or worse (11–13). In addition to empiric treatment with antibiotics, corticosteroids are intravitreally injected to blunt inflammation; however, final visual outcomes may remain poor, even with prompt medical intervention (7, 14).

The severity of *S. aureus* endophthalmitis can be attributed to its variety of secreted virulence factors. *S. aureus* regulates the expression of several virulence factors by the two-component quorum sensing system, accessory gene regulator (Agr), and its transcriptional regulator, staphylococcal accessory regulator (Sar), among others (15). The absence of Agr and Sar in *S. aureus* attenuated infection outcomes in an experimental rabbit model of *S. aureus* endophthalmitis (16, 17). Pore-forming toxins (PFTs) are a class of secreted virulence factors under the control of Agr. For *S. aureus,* PFTs include the alpha toxin and leukocidins (18, 19). These toxins have a similar mechanism that results in pore formation on the host target cell. PFT monomers bind onto their target receptor, resulting in oligomerization. Oligomers undergo conformational changes that allow the toxin to insert into the host cell membrane, ultimately leading to cation influx and lysis of the host cell (20, 21). The alpha toxin oligomers form a heptamer that targets a ubiquitously expressed receptor, a disintegrin and metalloprotease domain-containing protein 10 (ADAM10) (22). The *S. aureus* family of leukocidins include Panton Valentine Leukocidin (PVL), the gamma hemolysin, (HlgACB), leukocidin ED (LukED), and leukocidin AB/GH (LukAB/GH). Leukocidins consist of two subunits, the slow (S) and the fast (F), which combine to form an octamer (20). Leukocidins primarily target leukocytes by interacting with chemokine receptors or integrins; however, some leukocidins can target endothelial cells and erythrocytes (23).

Alpha toxin has been previously shown to contribute to retinal function decline in an experimental rabbit model of endophthalmitis (5), but this study did not report on intraocular bacterial burden or inflammation. Purified alpha toxin has been injected into the murine eye, resulting in the induction of proinflammatory cytokines and chemokines (IL-1β, TNF-α, and keratinocyte chemoattractant [KC]) but no neutrophil infiltration into the eye (24). It is unclear whether the amount of purified alpha toxin injected into the eye in that study was physiologically relevant. The role of leukocidins in the pathogenesis of endophthalmitis is not as clear. Injection of purified PVL and gamma toxin caused inflammation in the rabbit eye (25). PVL has also been shown to interact with retinal neurons, resulting in glial activation and an inflammatory response in an explanted retina model (26–28). These studies used purified leukocidin, and, as noted above, it is unclear whether these concentrations were physiologically relevant. Studies using isogenic mutants to investigate the role of gamma toxin (5, 29) and LukED (30) in the pathogenesis of *S. aureus* endophthalmitis reported no role for these toxins during infection. In the LukED study mentioned above, we reported that leukocidin subunits *lukSF-PV*, *lukED*, *lukGH*, and *hlgACB* were expressed in the mouse eye during infection (30). Given the similar mechanism of action between alpha toxin and the leukocidins, we sought to determine if these toxins collectively influenced the pathogenesis of *S. aureus* endophthalmitis.

We hypothesized that pore-forming toxins as a group contribute to the pathogenesis of *S. aureus* endophthalmitis. In the current study, we evaluated disease outcomes by intravitreally infecting mice with *S. aureus* strain LAC or mutants lacking all pore-forming toxins (LAC ΔΔΔΔΔ), lacking leukocidins only (LAC Δ*leukocidin*), or lacking alpha toxin only (LAC Δ*hla*). Our findings suggest that alpha toxin is the main virulence factor influencing the clinical pathogenesis of murine *S. aureus* endophthalmitis. Whether these findings translate to human disease is not known. In addition, detection of ADAM10^+^ neutrophils among infiltrating cells during endophthalmitis suggests that these cells may be potential targets for the alpha toxin.

## RESULTS

### *S. aureus* LAC and pore-forming toxin mutant derivatives exhibit similar growth kinetics *in vitro* and *ex vivo*

**Fig 1:**
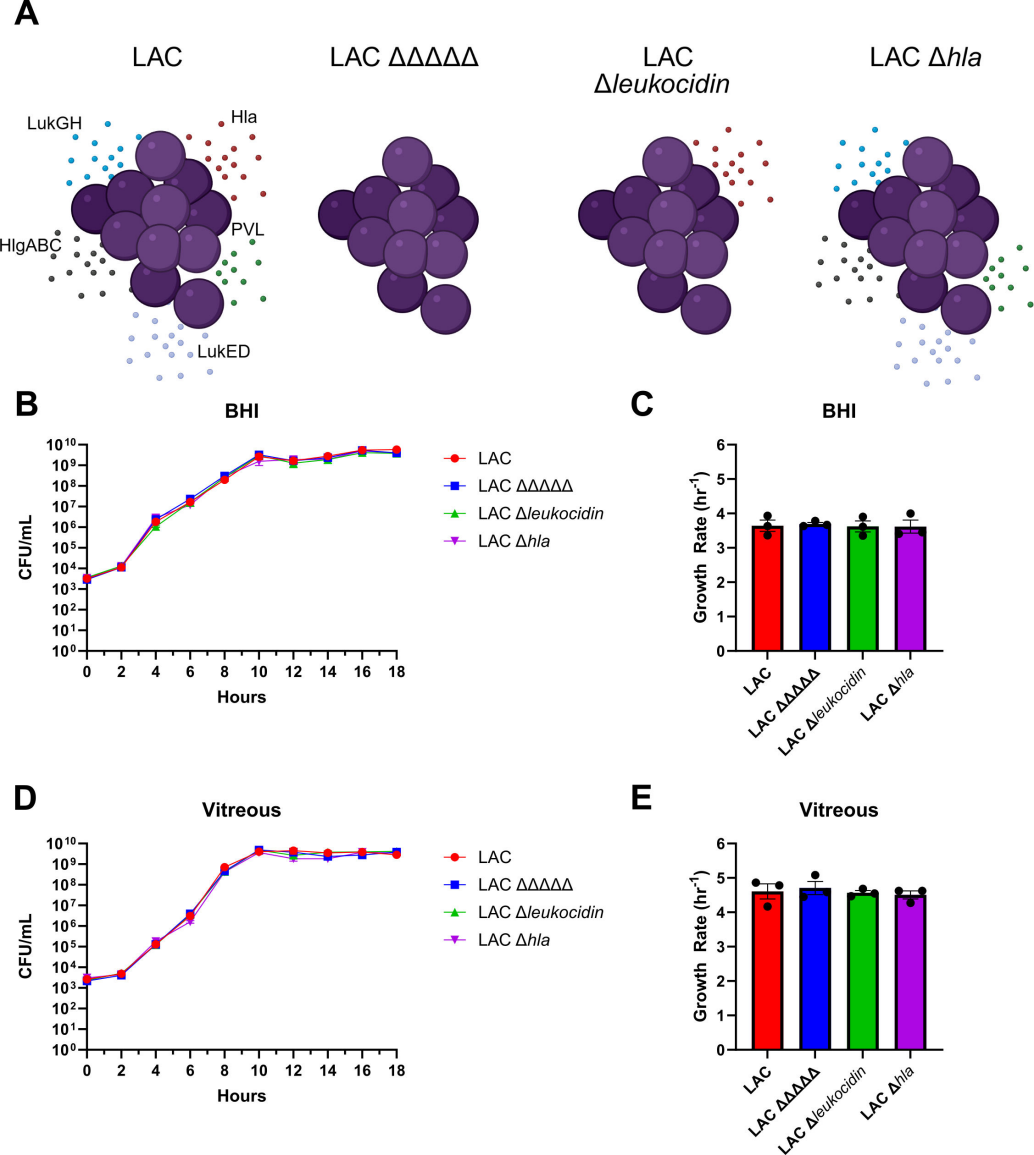
*S. aureus* LAC and PFT mutant derivatives exhibit similar growth kinetics *in vitro* and *ex vivo.* (**A**) Schematic diagram of strains used in this study, depicting the secreted toxins from *S. aureus* strain LAC and toxin-deficient mutants. The 18-h overnight cultures of *S. aureus* strains LAC, LAC ΔΔΔΔΔ, LAC Δ*leukocidin*, or LAC Δ*hla* were diluted to approximately 10^3^ CFU/mL in either BHI or explanted rabbit vitreous. Growth curves were generated in (**B**) BHI or (**D**) explanted rabbit vitreous. Growth rates were calculated during the exponential phase in (**C**) BHI or (**E**) explanted rabbit vitreous. All strains exhibited similar growth kinetics and growth rates in BHI and explanted rabbit vitreous. Data represent the mean ± SEM of three independent cultures. Statistical significance is indicated by *P* < 0.05 (Kruskal-Wallis test with Dunn’s multiple correction test).

The strains used in this study are shown in Table 1, and a schematic representation of secreted toxins is shown in Fig. 1A. Growth curves of *S. aureus* strain LAC, LAC ΔΔΔΔΔ, LAC Δ*leukocidin*, and LAC Δ*hla* were generated to compare growth kinetics. Figure 1B shows that LAC and all mutants grew to similar concentrations in BHI (*P* > 0.05 at all time points). All strains reached the stationary phase at 10 h. To assess if the strains exhibited differences in growth kinetics, the average growth rate during the exponential phase was calculated for each strain (Fig. 1C). No differences in the growth rate were observed between LAC or LAC ΔΔΔΔΔ (*P* > 0.9999), LAC Δ*leukocidin* (*P* > 0.9999), or LAC Δ*hla* (*P* > 0.9999). Figure 1D depicts *ex vivo* growth curves, where all strains were cultured in explanted vitreous to simulate the intraocular environment. LAC and all mutants grew to similar concentrations in vitreous (*P* > 0.05 at all time points), reaching the stationary phase at 10 h. Figure 1E depicts the average growth rates of these strains during the exponential phase in explanted vitreous. No differences in the growth rate were observed between LAC or LAC ΔΔΔΔΔ (*P* > 0.9999), LAC Δ*leukocidin* (*P* > 0.9999), or LAC Δ*hla* (*P* > 0.9999). These results showed that LAC and all mutants exhibited similar growth kinetics *in vitro* and *ex vivo*, suggesting that any differences that might be observed *in vivo* were independent of bacterial growth rates.

**TABLE 1:** Bacterial strains used in this study

Strain	Mutations	Toxin disrupted	Reference
LAC	Wild-type	None	(31)
LAC ΔΔΔΔΔ	∆*lukGH* ∆*hlgACB::tet* ∆*lukED::kan* ∆*pvl::spec* ∆*hla::erm*	LukGH, HlgACB, LukED, PVL, and Hla	(31)
LAC Δ*leukocidin*	∆*lukGH* ∆*hlgACB::tet* ∆*lukED::kan* ∆*pvl::spec*	LukGH, HlgACB, LukED, and PVL	(31)
LAC Δ*hla*	Δ*hla*	Hla	(31)

### Pore-forming toxins are expressed in explanted rabbit vitreous

**Fig 2:**
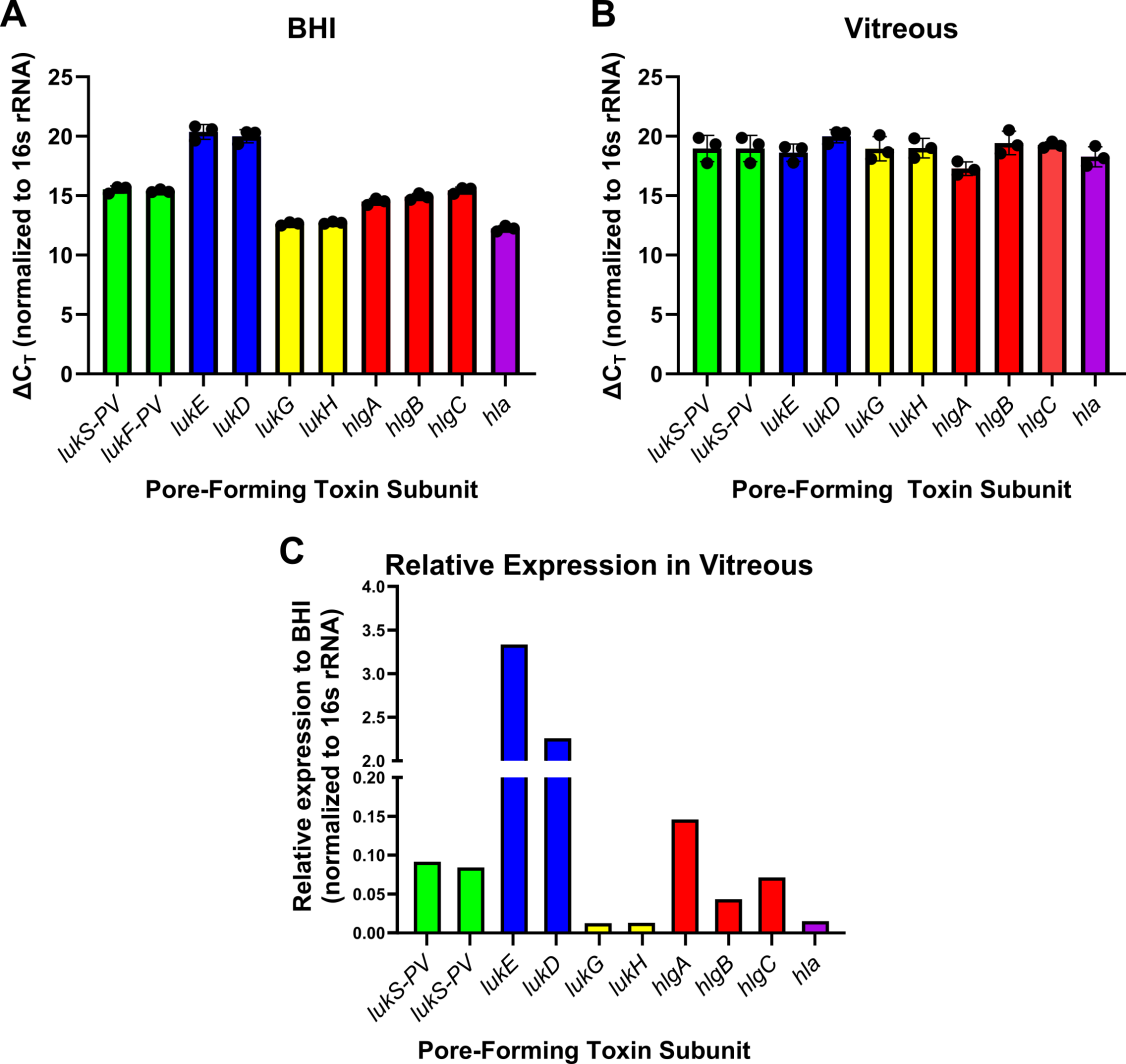
PFTs are expressed in explanted rabbit vitreous. *S. aureus* strain LAC was cultured in BHI or explanted rabbit vitreous. After 18 h, RNA was isolated, and qRT-PCR was performed to determine PFT expression in both environments. 16s rRNA was used a control. (**A,B**) ΔC_T_ values of PFT subunit genes when *S. aureus* LAC was cultured in (**A**) BHI or (**B**) explanted rabbit vitreous. (**C**) Fold-change expression of PFT subunit genes in explanted rabbit vitreous relative to BHI. Transcript levels of *lukE* and *lukD* were three-fold and two-fold greater in vitreous rabbit relative to BHI, respectively. Expression of the other PFT subunits was greater in BHI relative to that in vitreous. Data are representative of mean ΔC_T_ ± SEM of *n* = 3 and mean fold change across three independent experiments.

To quantify the expression of PFTs in the intraocular environment, *S. aureus* LAC was cultured in explanted rabbit vitreous, and the expression of PFTs was compared to that of culturing in BHI. Because PFTs are under the control of Agr-directed quorum sensing, the expression was assessed at 18 h post-inoculation. Figure 2A depicts the ΔC_T_ values of PFTs in the 18-h BHI culture, while Fig. 2B shows the ΔC_T_ values of PFTs in the 18-h explanted rabbit vitreous culture. PFTs were expressed in both environments. Figure 2C depicts the relative expression of PFTs in vitreous cultures compared to BHI cultures. In general, we observed that PFT expression in vitreous was less than that in BHI, with the exception of *lukE* and *lukD*, where the expression was three- and two-fold greater in vitreous than in BHI, respectively.

### Effect of pore-forming toxins on anterior segment pathology

**Fig 3:**
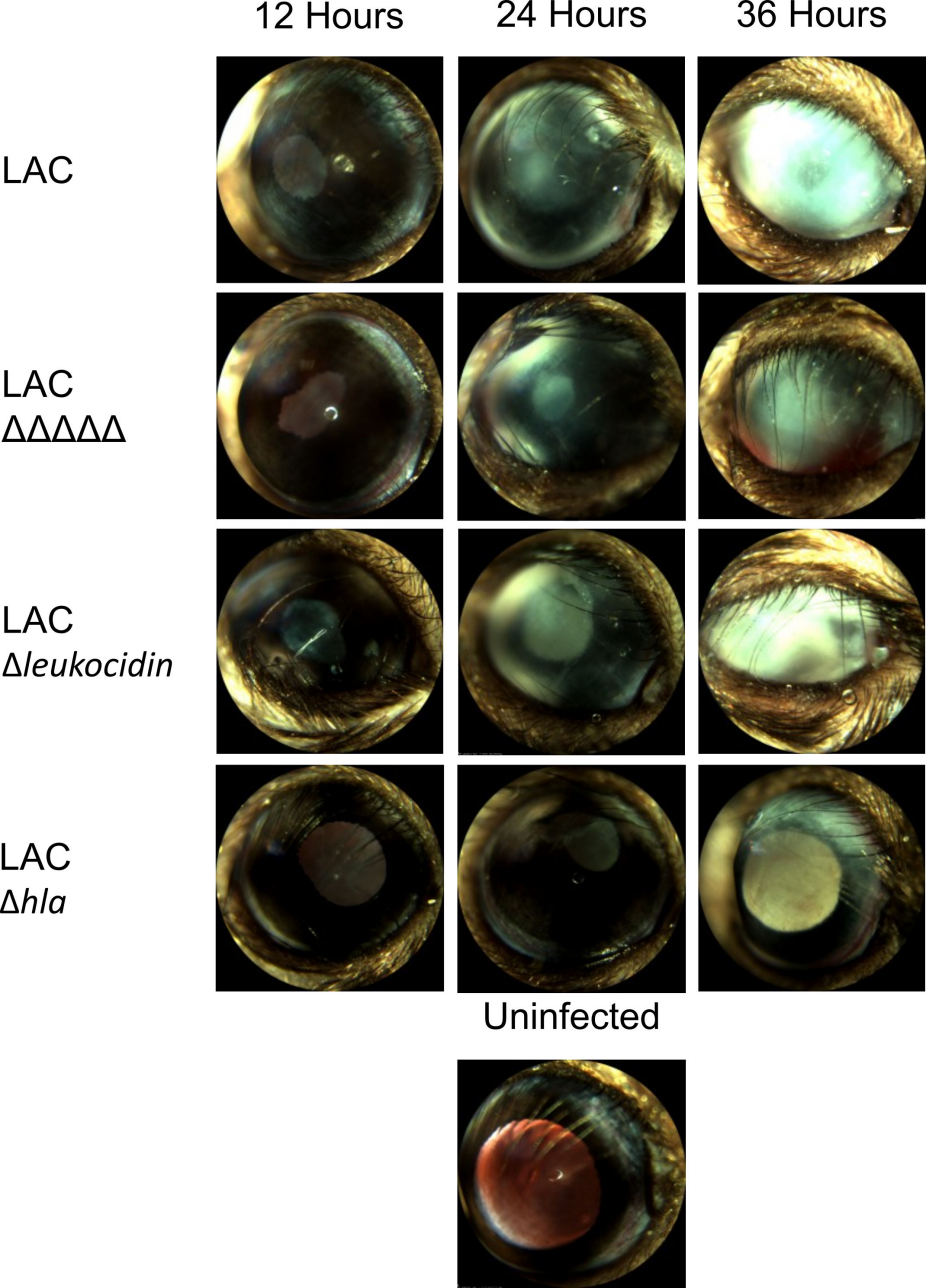
Effect of PFTs on anterior segment pathology**.** Eyes of C57BL6/J mice were infected with 5,000 CFU of *S. aureus* strains LAC, LAC ΔΔΔΔΔ, LAC Δ*leukocidin*, or LAC Δ*hla*. Prior to imaging, mice were anesthetized with isoflurane, and topical drops were administered for dilation and anesthesia. Eyes were imaged on the Micron IV at 12, 24, or 36 h post infection. Anterior segment inflammation evolved as the infection progressed regardless of the infecting strains. Infections with LAC ΔΔΔΔΔ or LAC Δ*hla* exhibited attenuated inflammation relative to infections with LAC or LAC Δ*leukocidin*. An uninfected eye is shown as a reference. Images are representative of *n* = 3–5 eyes per time point.

To determine whether the absence of PFTs affected anterior segment pathology during endophthalmitis, eyes of mice infected with LAC, LAC ΔΔΔΔΔ, LAC Δ*leukocidin*, or LAC Δ*hla* were evaluated by slit-lamp microscopy at 12, 24, or 36 h post-infection (Fig. 3). An uninfected eye is shown for reference. Uninfected eyes had no anterior segment inflammation, a visibly dilated pupil, and a clear view through to the posterior segment. At 12 h post-infection, eyes infected with LAC, LAC ΔΔΔΔΔ, LAC Δ*leukocidin*, or LAC Δ*hla* exhibited minimal anterior segment inflammation and dilation defects, limiting the view through to the posterior segment. At 24 h post-infection, anterior segment inflammation was more visible in eyes infected with LAC, and infected eyes became completely opaque by 36 h post-infection. At 24 h post-infection, eyes infected with LAC ΔΔΔΔΔ exhibited anterior segment inflammation, although this inflammation was less pronounced compared to that in eyes infected with LAC at the same time point. Inflammation continued to be less pronounced in eyes infected with LAC ΔΔΔΔΔ at 36 h. Infections with LAC Δ*leukocidin* evolved similarly as eyes infected with LAC, resulting in completely opaque eyes by 36 h post-infection. Similar to eyes infected with LAC ΔΔΔΔΔ, eyes infected with LAC Δ*hla* exhibited less anterior segment inflammation at both 24 h and 36 h post-infection compared to eyes infected with LAC. Collectively, these results demonstrated that although all strains replicated similarly in the mouse eye, the absence of all PFTs or alpha toxin alone resulted in slower-evolving intraocular inflammation.

### Effect of pore-forming toxins on ocular pathology

**Fig 4:**
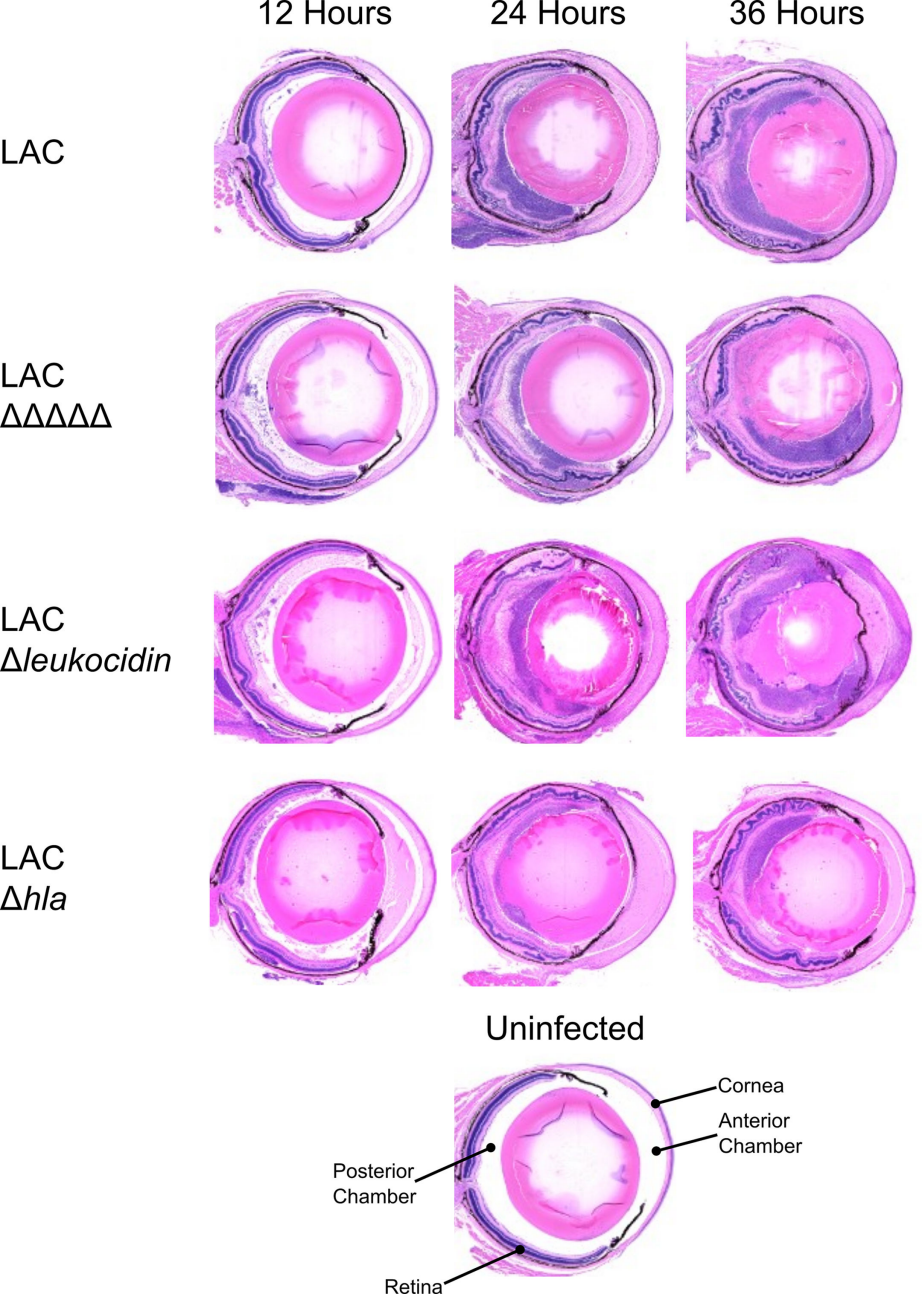
Effect of PFTs on ocular pathology. Eyes of C57BL6/J mice were infected with 5,000 CFU of *S. aureus* strain LAC, LAC ΔΔΔΔΔ, LAC Δ*leukocidin*, or LAC Δ*hla*. Eyes were harvested at 12, 24, or 36 h post-infection and incubated in Perfix for 24 h and then transferred to 70% ethanol prior to processing. Eyes were stained with hematoxylin and eosin. Regardless of the infecting strain, all eyes exhibited inflammation, deposition of fibrin, and damage to the retina. Infection in eyes infected with LAC ΔΔΔΔΔ or LAC Δ*hla* evolved slower compared to infection in eyes infected with LAC and LAC Δ*leukocidin*. An uninfected eye is shown as a control. Sections are representative of *n* = 3–5 eyes per time point.

To determine whether the absence of PFTs affected retinal pathology, histological changes were evaluated in mice infected with LAC, LAC ΔΔΔΔΔ, LAC Δ*leukocidin*, or LAC Δ*hla* at 12, 24, or 36 h post-infection (Fig. 4). An uninfected eye is shown for reference. Uninfected eyes had intact retinal architecture, no intraocular inflammation or fibrin, and clear corneas. At 12 h post-infection, eyes infected with LAC had infiltration of inflammatory cells, deposition of fibrin in the anterior and posterior chambers, and damage to the retinal architecture. By 24 and 36 h post-infection, these pathological features continued to evolve along with the presence of corneal inflammation and edema. At 12 h post-infection, the pathological features in eyes infected with LAC ΔΔΔΔΔ, LAC Δ*leukocidin*, or LAC Δ*hla* were comparable to that of eyes infected with LAC. However, infections with LAC ΔΔΔΔΔ or LAC Δ*hla* evolved more slowly. While eyes infected with LAC ΔΔΔΔΔ or LAC Δ*hla* exhibited inflammation in the posterior and anterior chambers, deposition of fibrin, corneal edema, and damage to the retina, these pathological features were less severe in eyes infected with LAC. Infections in eyes infected with LAC Δ*leukocidin* evolved similarly to that of eyes infected with LAC at all time points. Collectively, these results demonstrated that the absence of all PFTs or alpha toxin alone attenuated evolving ocular pathology during endophthalmitis in a mouse infection model.

### Effect of pore-forming toxins on retinal function

**Fig 5:**
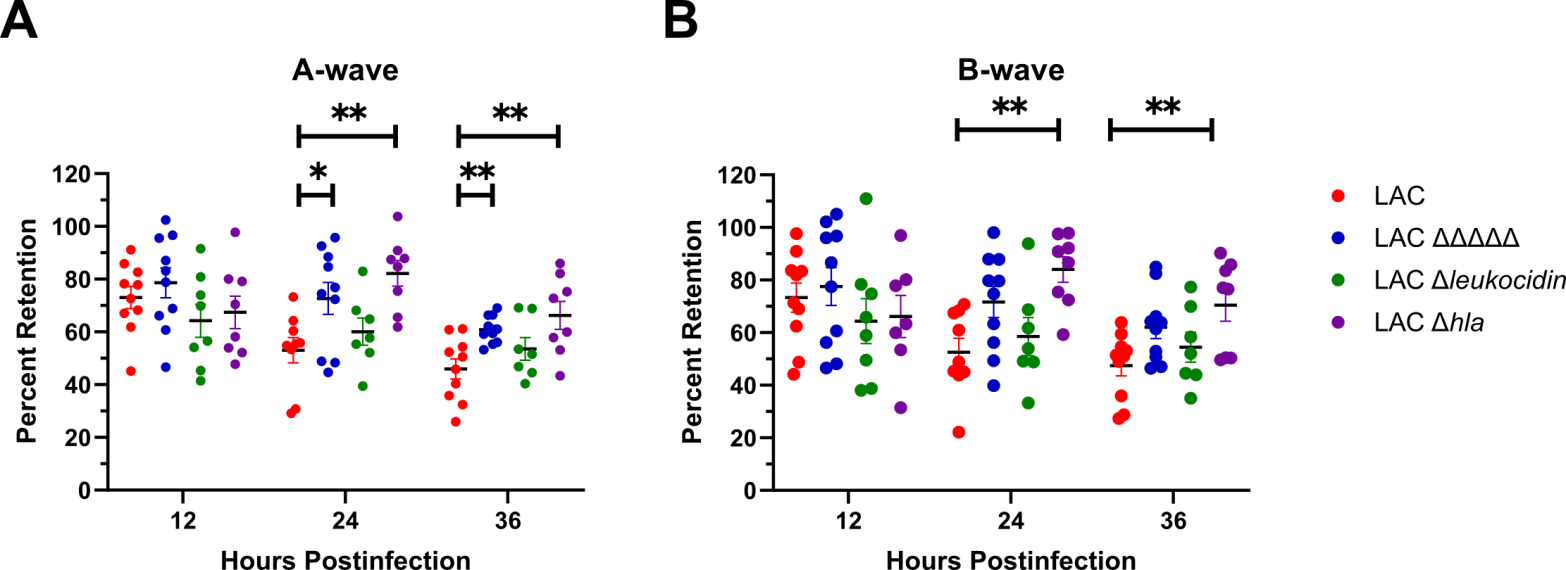
Effect of PFTs on retinal function**.** Eyes of C57BL6/J mice were infected with *S. aureus* strain LAC, LAC ΔΔΔΔΔ, LAC Δ*leukocidin*, or LAC Δ*hla*. At 12, 24, or 36 h post-infection, retinal function was analyzed by ERG. Retinal function decline was observed in all infected eyes throughout the course of infection. (**A**) Eyes infected with LAC ΔΔΔΔΔ or LAC Δ*hla* exhibited greater A-wave responses compared to the eyes infected with LAC at both 24 and 36 h post-infection. No differences in A-wave retention were observed between the eyes infected with LAC or LAC Δ*leukocidin*. (**B**) Eyes infected with LAC Δ*hla* exhibited a greater B-wave response compared to the eyes infected with LAC at 24 and 36 h post-infection. The eyes infected with LAC ΔΔΔΔΔ exhibited a trend of greater B-wave response compared to the eyes infected with LAC at 24 and 36 h post-infection, but these differences were not significant. No differences in B-wave retention were observed between the eyes infected with LAC or LAC Δ*leukocidin*. Data represent the mean percent retention ± SEM of *n* = 7–10 mice/group. Statistical significance is indicated by *P < 0.05; **P < 0.01; ***P < 0.001 (one-way ANOVA with Dunnett’s multiple comparisons test).

To determine whether the absence of PFTs affected retinal function, we utilized electroretinography to compare the percent retention of the A-wave and B-wave response of eyes infected with LAC, LAC ΔΔΔΔΔ, LAC Δ*leukocidin*, or LAC Δ*hla* at 12, 24, or 36 h post-infection. Figure 5A depicts the percent retention of the A-wave response of infected eyes. At 12 h post-infection, eyes infected with LAC, LAC ΔΔΔΔΔ (*P* = 0.8009), LAC Δ*leukocidin* (*P* = 0.5520), or LAC Δ*hla* (*P* = 0.8218) exhibited similar percent A-wave retention. At 24 h, eyes infected with LAC ΔΔΔΔΔ (*P* = 0.0288) or LAC Δ*hla* (*P* = 0.0018) exhibited a greater percent retention in the A-wave response compared to eyes infected with LAC. At 36 h post-infection, eyes infected with LAC ΔΔΔΔΔ (*P* = 0.0160) or LAC Δ*hla* (*P* = 0.0017) exhibited a greater percent retention in the A-wave response compared to eyes infected with LAC. No differences in the A-wave response were observed between eyes infected with LAC and LAC Δ*leukocidin* at 24 h (*P* = 0.7108) or 36 h (*P* = 0.4006) post-infection.

Figure 5B depicts the percent retention of the B-wave response of infected mice. At 12 h, eyes infected with LAC, LAC ΔΔΔΔΔ (*P* = 0.9487), LAC Δ*leukocidin* (*P* = 0.7227), or LAC Δ*hla* (*P* = 0.0.8459) exhibited a similar percent retention of the B-wave response. The B-wave percent retention was greater in eyes infected with LAC ΔΔΔΔΔ at 24 h (*P* = 0.0539) and at 36 h (*P* = 0.0838) compared to eyes infected with LAC, but these differences were not statistically significant. No differences were observed in the B-wave percent retention between eyes infected with LAC or LAC Δ*leukocidin* at 24 h (*P* = 0.8324) or 36 h (*P* = 0.6565) post-infection. Eyes infected with LAC Δ*hla* exhibited a greater B-wave percent retention at 24 h (*P* = 0.0019) and 36 h (*P* = 0.0063) post-infection compared to eyes infected with LAC. These results demonstrated that the absence of all PFTs or the absence of the alpha toxin alone preserved retinal function. Collectively, these results suggest that the alpha toxin is a key contributor to ocular pathology and retinal function loss during *S. aureus* endophthalmitis.

### Effect of pore-forming toxins on intraocular burden

**Fig 6:**
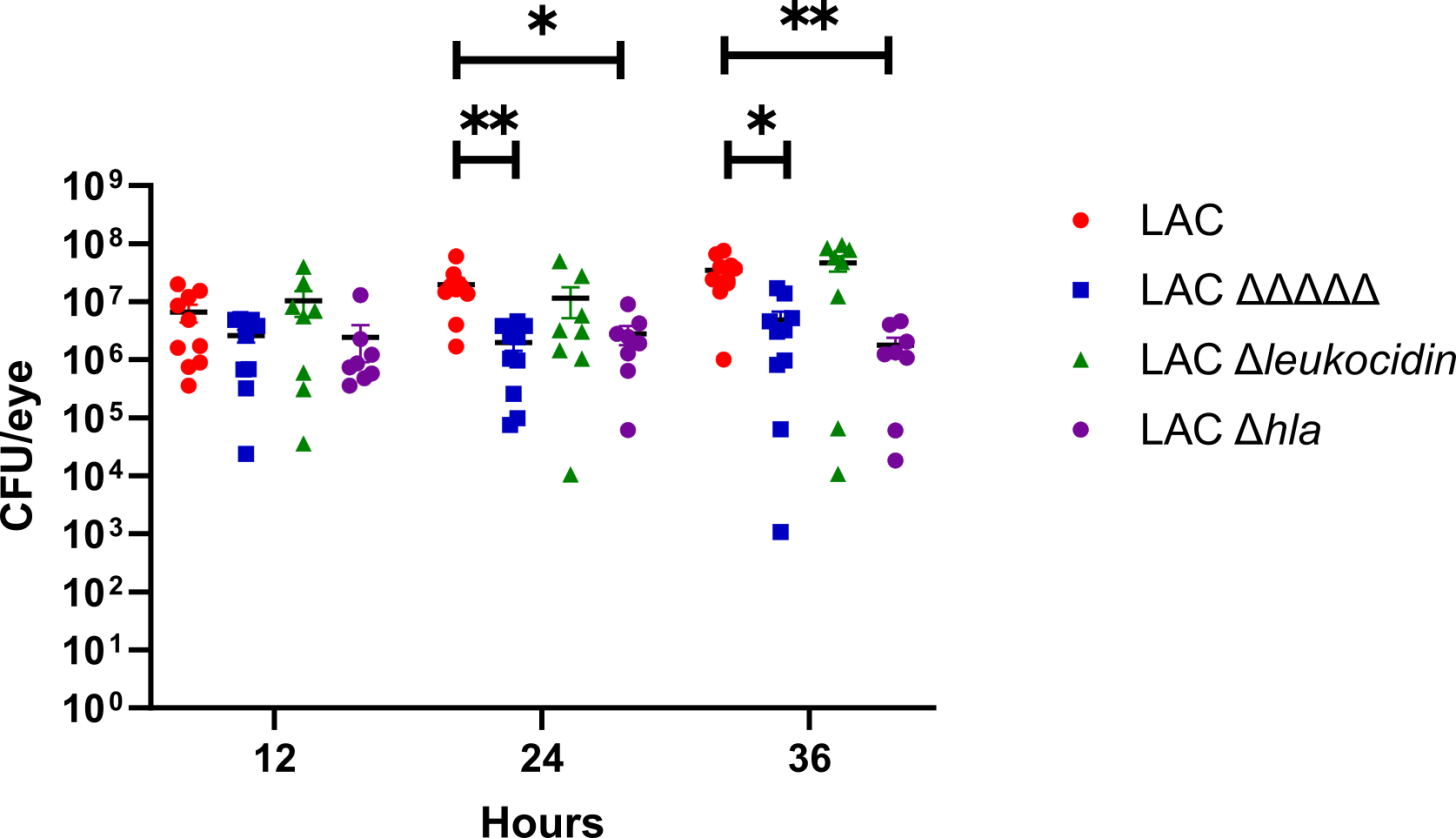
Effect of PFTs on intraocular burden**.** Eyes of C57BL6/J mice were infected with *S. aureus* strains LAC, LAC ΔΔΔΔΔ, LAC Δ*leukocidin*, or LAC Δ*hla*. Eyes were harvested and homogenized at 12, 24, or 36 h post-infection, and homogenates were plated on BHI plates to quantify intraocular CFUs. Eyes infected with LAC ΔΔΔΔΔ or LAC Δ*hla* exhibited less intraocular CFUs compared to eyes infected with LAC at 24 and 36 h post-infection. No differences in intraocular CFUs were observed at any time point between eyes infected with LAC or LAC Δ*leukocidin*. Data represent mean CFU ± SEM of *n* = 8–10 mice/group. Statistical significance is indicated by *P < 0.05; **P < 0.01; ***P < 0.001 (Kruskal-Wallis test with Dunn’s multiple correction test).

To determine whether the absence of PFTs affected the growth of *S. aureus* in the eye, we compared intraocular CFUs of eyes infected with LAC, LAC ΔΔΔΔΔ, LAC Δ*leukocidin*, or LAC Δ*hla*, at 12, 24, or 36 h post-infection (Fig. 6). At 12 h, eyes infected with LAC, LAC ΔΔΔΔΔ (*P* = 0.6430), LAC Δ*leukocidin* (*P* > 0.9999), or LAC Δ*hla* (*P* = 0.3399) contained similar numbers of CFU/eye. At 24 h post-infection, we observed greater CFU/eye in eyes infected with LAC compared to eyes infected with LAC ΔΔΔΔΔ (*P* = 0.0023) or LAC Δ*hla* (*P* = 0.0143). Numbers of CFU/eye were also greater in eyes infected with LAC compared to eyes infected with LAC ΔΔΔΔΔ (*P* = 0.0247) or LAC Δ*hla* (*P* = 0.0099) at 36 h post-infection. No differences were observed in numbers of CFU/eye infected with LAC or LAC Δ*leukocidin* at 24 h post-infection (*P* = 0.1892) or 36 h post-infection (*P* > 0.9999). These results demonstrated that the absence of alpha toxin led to reduced bacterial burden, which may have resulted in less ocular pathology and preservation of retinal function during *S. aureus* endophthalmitis.

### Effect of pore-forming toxins on intraocular inflammation

**Fig 7:**
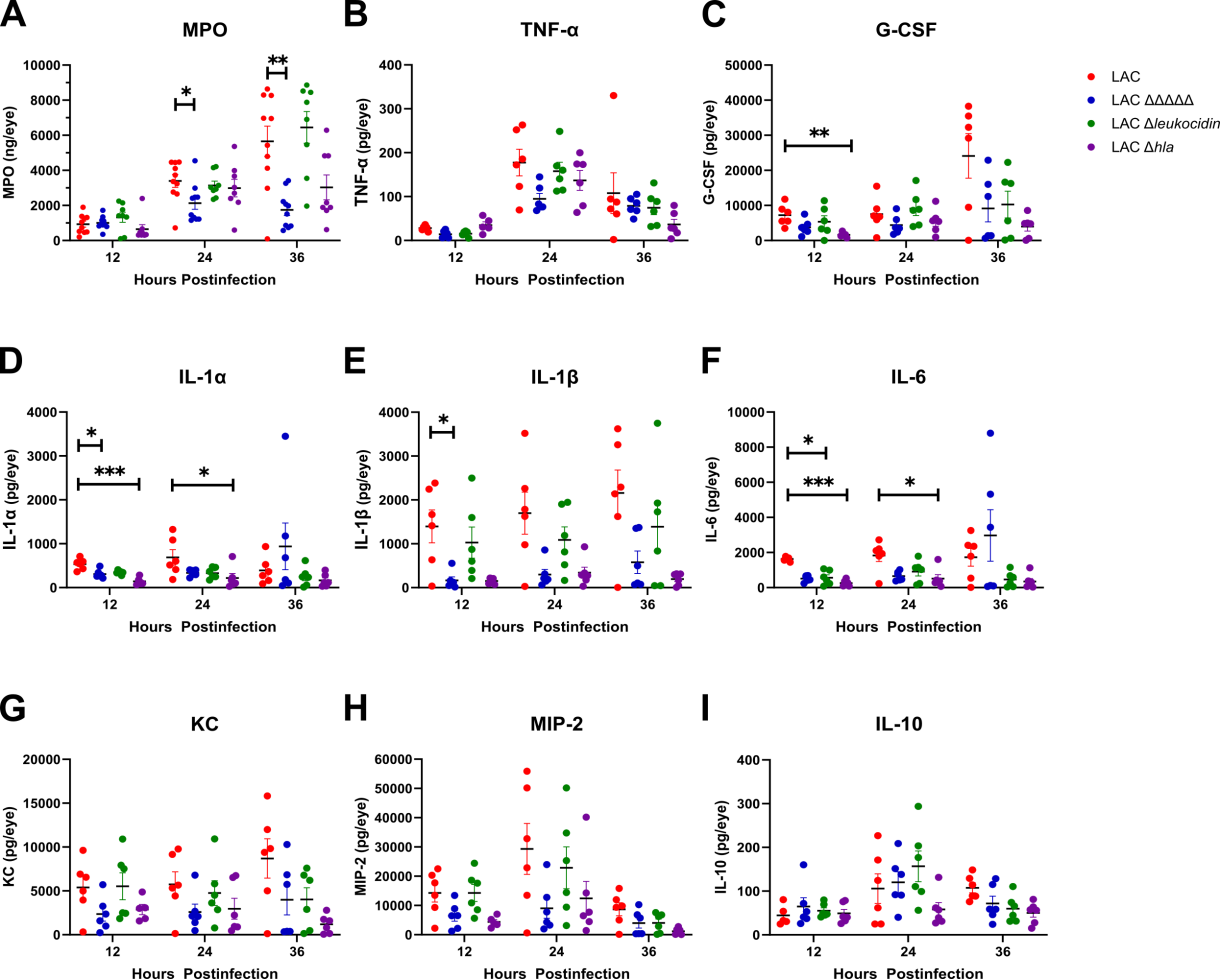
Effect of PFTs on intraocular inflammation. Eyes of C57BL6/J mice were infected with *S. aureus* strains LAC, LAC ΔΔΔΔΔ, LAC Δ*leukocidin*, or LAC Δ*hla*. Eyes were harvested and homogenized at 12, 24, or 36 h post-infection, and homogenates were used to analyze inflammation via enzyme-linked immunosorbent assay (ELISA) or Bio-Plex. (**A**) MPO levels were quantified by sandwich ELISA. Eyes infected with LAC ΔΔΔΔΔ exhibited less intraocular MPO compared to eyes infected with LAC at 24 and 36 h post-infection. (**B**) TNF-α, (**C**) G-CSF, (**D**) IL-1α, (**E**) IL-1β, (**F**) IL-6, (**G**) KC, (**H**) MIP-2, and (**I**) IL-10 were quantified by Bio-Plex. No differences in TNF-α, KC, MIP-2, or IL-10 were detected in eyes infected with LAC, LAC ΔΔΔΔΔ, LAC Δ*leukocidin*, or LAC Δ*hla* at any time point. Eyes infected with LAC ΔΔΔΔΔ exhibited less IL-1α, IL-1β, and IL-6 at 12 h post-infection compared to that of LAC-infected eyes. Infection with LAC Δ*hla* resulted in less G-CSF, IL-1α, and IL-6 at 12 h post-infection, and less IL-1α and IL-6 at 24 h post-infection, relative to that of eyes infected with LAC. MPO data represent mean MPO (ng/eye) ± SEM of *n* = 8–10 eyes/group. Bio-Plex data represent mean cytokine/chemokine concentrations (ng/eye) ± SEM of *n* = 5–6 eyes/group. Statistical significance is indicated by *P < 0.05; **P < 0.01; ***P < 0.001. Data were analyzed based on the distribution (Kruskal-Wallis test with Dunn’s multiple correction test).

To determine whether the absence of PFTs affected intraocular inflammation during infection, we quantified myeloperoxidase (MPO), cytokines, and chemokines in eyes infected with LAC, LAC ΔΔΔΔΔ, LAC Δ*leukocidin*, or LAC Δ*hla*, at 12, 24, or 36 h post-infection (Fig. 7). Figure 7A shows the abundance of MPO in infected eyes. At 12 h post-infection, there were no differences in mean MPO/eye in eyes infected with LAC, LAC ΔΔΔΔΔ (*P* = 0.4619), LAC Δ*leukocidin* (*P* = 0.9154), or LAC Δ*hla* (*P* > 0.9999). At 24 h, eyes infected with LAC had greater mean MPO/eye compared to eyes infected with LAC ΔΔΔΔΔ (*P* = 0.0326) but similar mean MPO/eye compared to eyes infected with LAC Δ*leukocidin* (*P* > 0.9999) or LAC Δ*hla* (*P* = 0.8330). At 36 h post-infection, eyes infected with LAC continued to have greater mean MPO/eye compared to eyes infected with LAC ΔΔΔΔΔ (*P* = 0.0102). There were no statistically significant differences in mean MPO/eye in eyes infected with LAC and LAC Δleukocidin (*P* > 0.9999). At 36 h post-infection, eyes infected with LAC Δhla (*P* = 0.1861) had less intraocular MPO compared to eyes infected with LAC, but this difference was not statistically significant.

Figure 7B shows the abundance of TNF-α in infected eyes. At 12 h post-infection, eyes infected with LAC ΔΔΔΔΔ (*P* = 0.0610) or LAC Δ*leukocidin* (*P* = 0.0805) had less intraocular TNF-α compared to eyes infected with LAC, but these differences were not statistically significant. There were no statistical differences in intraocular TNF-α in eyes infected with LAC or LAC Δ*hla* (*P* > 0.9999) at 12 h post-infection. At 24 h post-infection, eyes infected with LAC ΔΔΔΔΔ (*P* = 0.0666) had less TNF-α compared to eyes infected with LAC, but these differences were not statistically significant. At this time point, no statistical differences in intraocular TNF-α were detected in eyes infected with LAC, LAC Δ*leukocidin* (*P* > 0.9999), or LAC Δ*hla* (*P* > 0.9999). Similarly, no statistical differences in intraocular TNF-α were observed at 36 h post-infection in eyes infected with LAC, LAC ΔΔΔΔΔ (*P* > 0.9999), or LAC Δ*leukocidin* (*P* > 0.9999). However, at 36 h post-infection, eyes infected with LAC Δ*hla* (*P* = 0.1986) had less intraocular TNF-α compared to eyes infected with LAC, but this difference was not significant.

Figure 7C shows the abundance of G-CSF of infected eyes. At 12 h post-infection, there was no difference in intraocular G-CSF among eyes infected with LAC, LAC ΔΔΔΔΔ (*P* = 0.2660), or LAC Δ*leukocidin* (*P* = 0.9210); however, there was a decrease of intraocular G-CSF in eyes infected with LAC Δ*hla* (*P* = 0.0070) compared to eyes infected with LAC at 12 h post-infection. At 24 h, there was no difference in intraocular G-CSF among eyes infected with LAC, LAC ΔΔΔΔΔ (*P* = 0.5337), LAC Δ*leukocidin* (*P* > 0.9999), or LAC Δ*hla* (*P* > 0.9999). Similarly, there was no difference in intraocular G-CSF among eyes infected with LAC, LAC ΔΔΔΔΔ (*P* = 0.4591), LAC Δ*leukocidin* (*P* = 0.6170), or LAC Δ*hla* (*P* > 0.0537) at 36 h post-infection. At 36 h post-infection, eyes infected with LAC Δ*hla* had less intraocular G-CSF compared to eyes infected with LAC, but this difference was not statistically significant.

Figure 7D shows the abundance of IL-1α in infected eyes. At 12 h post-infection, eyes infected with LAC exhibited greater intraocular IL-1α compared to eyes infected with LAC ΔΔΔΔΔ (*P* = 0.0320) or LAC Δ*hla* (*P* = 0.0003). There was no difference in intraocular IL-1α between eyes infected with LAC or LAC Δ*leukocidin* (*P* = 0.2429). At 24 h post-infection, eyes infected with LAC exhibited greater intraocular IL-1α than eyes infected with LAC Δ*hla* (*P* = 0.0112), but no statistically significant differences were observed in intraocular IL-1α compared to eyes infected with LAC ΔΔΔΔΔ (*P* = 0.4249) or LAC Δ*leukocidin* (*P* = 0.3927). At 36 h post-infection, there were no differences in intraocular IL-1α in eyes infected with LAC, LAC ΔΔΔΔΔ (*P* > 0.9999), LAC Δ*leukocidin* (*P* > 0.9999), or LAC Δ*hla* (*P* = 0.5337).

Figure 7E shows the abundance of IL-1β in infected eyes. At 12 h post-infection, eyes infected with LAC exhibited greater intraocular IL-1β compared to eyes infected with LAC ΔΔΔΔΔ (*P* = 0.0283). No differences in intraocular IL-1β were observed in eyes infected with LAC, LAC Δ*leukocidin* (*P* > 0.9999), or LAC Δ*hla* (*P* = 0.1298). At 24 h post-infection, eyes infected with LAC ΔΔΔΔΔ (*P* = 0.0667) or LAC Δ*hla* (*P* = 0.1364) had less intraocular IL-1β compared to eyes infected with LAC, but these differences were not statistically significant. At this time point, no statistical differences in intraocular IL-1β were observed in eyes infected with LAC or LAC Δ*leukocidin* (*P* > 0.9999). At 36 h post-infection, eyes infected with LAC ΔΔΔΔΔ (*P* = *P* = .3340) or LAC Δ*hla* (*P* = 0.3340) had less intraocular IL-1β compared to eyes infected with LAC, but these differences were not statistically significant. No differences in intraocular IL-1β were observed in eyes infected with LAC or LAC Δ*leukocidin* (*P* > 0.9999) at 36 h post-infection.

Figure 7F shows the abundance of IL-6 in infected eyes. At 12 h post-infection, eyes infected with LAC exhibited greater intraocular IL-6 compared to eyes infected with LAC Δ*leukocidin* (*P* = 0.0356) or LAC Δ*hla* (*P* = 0.0008). Eyes infected with LAC ΔΔΔΔΔ (*P* = 0.0768) had reduced IL-6 compared to eyes infected with LAC at 12 h post-infection, but this difference was not statistically significant. Similarly, at 24 h post-infection, eyes infected with LAC ΔΔΔΔΔ (*P* = 0.1812) had reduced IL-6 compared to eyes infected with LAC, but this difference was not statistically significant. Eyes infected with LAC had statistically greater intraocular IL-6/eye compared to eyes infected with LAC Δ*hla (P* = 0.0212) at 24 h post-infection. No statistical differences were observed among eyes infected with LAC or LAC Δ*leukocidin* (*P* = 0.3074) at this time point. At 36 h post-infection, no differences in intraocular IL-6 were observed in eyes infected with LAC, LAC ΔΔΔΔΔ (*P* > 0.9999), LAC Δ*leukocidin* (*P* = 0.7093), or LAC Δ*hla* (*P* = 0.3927).

Figure 7G through H shows the abundance of KC and MIP-2 in infected eyes, respectively. We observed less intraocular KC in eyes infected with LAC ΔΔΔΔΔ at 12 (*P* = 0.1833), 24 (*P* = 0.4954), and 36 (*P* = 0.6170) h post-infection compared to eyes infected with LAC, but these differences were not statistically significant. Similarly, we observed less intraocular KC in eyes infected with LAC Δ*hla* at 12 (*P* = 0.5739), 24 (*P* = 0.6170), and 36 (*P* = 0.0599) h post-infection compared to eyes infected with LAC, but these differences were not statistically significant. No differences were observed in intraocular KC in eyes infected with LAC or LAC Δ*leukocidin* at 12 (*P* > 0.9999), 24 (*P* > 0.9999), or 36 (*P* = 0.7590) h post-infection. We observed less intraocular MIP-2 in eyes infected with LAC ΔΔΔΔΔ at 12 (*P* = 0.1664), 24 (*P* = 0.2592), and 36 (*P* = 0.6170) h post-infection compared to eyes infected with LAC, but these differences were not statistically significant. Similarly, we observed less intraocular KC in eyes infected with LAC Δ*hla* at 12 (*P* = 0.0663), 24 (*P* = 0.4954), and 36 (*P* = 0.0599) h post-infection compared to eyes infected with LAC, but these differences were not statistically significant. No differences were observed in intraocular KC in eyes infected with LAC or LAC Δ*leukocidin* at 12 (*P* > 0.9999), 24 (*P* > 0.9999), or 36 (*P* = 0.7590) h post-infection.

Figure 7I shows intraocular IL-10 during infection. While not a proinflammatory cytokine, we assessed intraocular IL-10 as this cytokine contributes to ocular immune privilege (32). At 12 h post-infection, no differences in intraocular IL-10 were observed in eyes infected with LAC, LAC ΔΔΔΔΔ (*P* > 0.9999), LAC Δ*leukocidin* (*P* = 0.8090), or LAC Δ*hla* (*P* > 0.9999). Similarly, at 24 and 36 h, no differences were detected in eyes infected with LAC, LAC ΔΔΔΔΔ (24 h *P* > 0.9999; 36 h *P* = 0.8251), LAC Δ*leukocidin* (24 h *P* = 0.6848; 36 h *P* = 0.4432), or LAC Δ*hla* (24 h *P* = 0.9809; 36 h *P* = 0.2205).

These results demonstrated that eyes infected with PFT-deficient mutants (namely, alpha toxin) exhibited less intraocular MPO, G-CSF, IL-1α, IL-1β, and IL-6, which further suggests that the presence of alpha toxin contributes to shaping the intraocular inflammatory response during *S. aureus* endophthalmitis.

### Effect of pore-forming toxins on neutrophil infiltration

**Fig 8:**
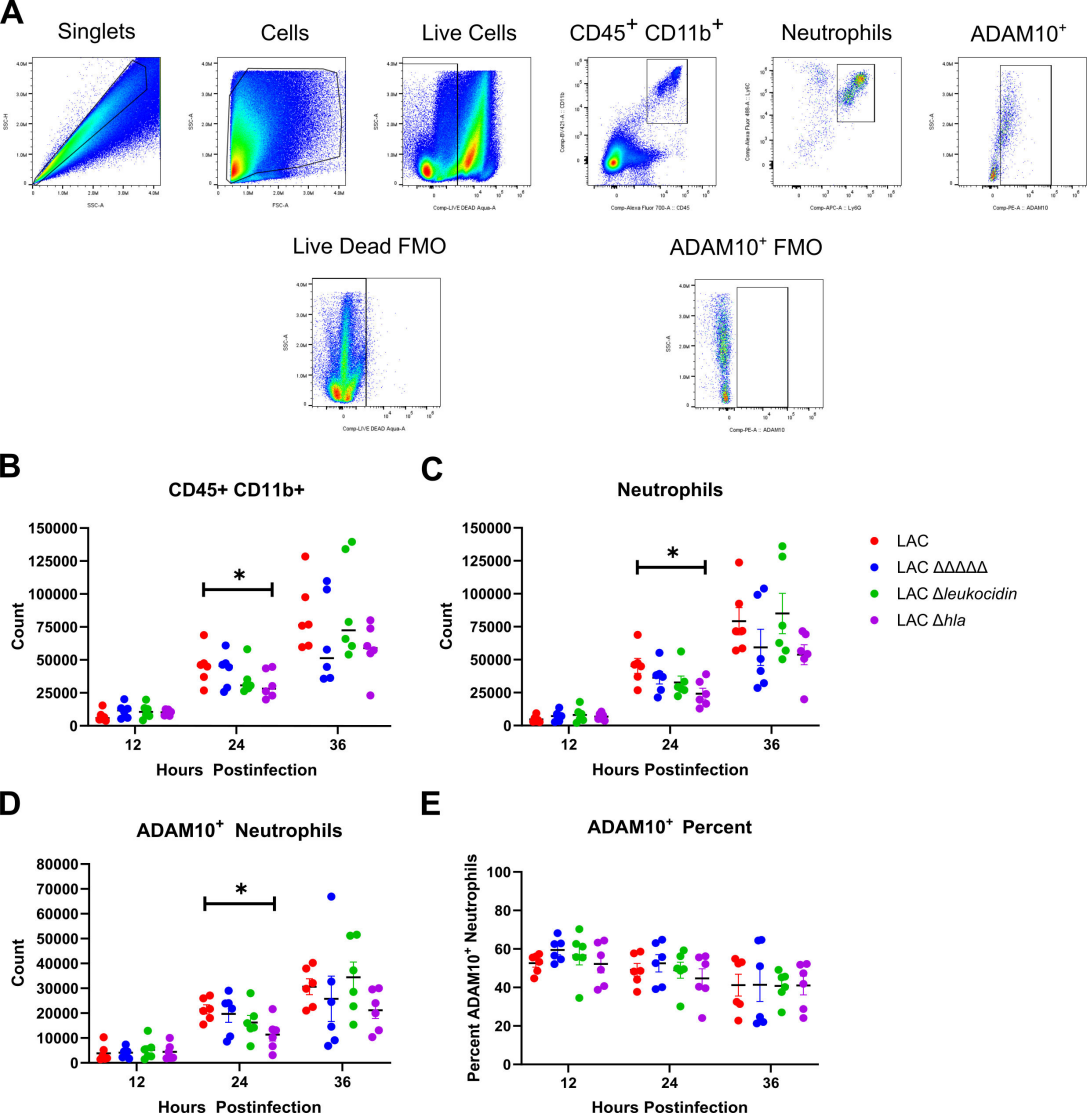
Effect of PFTs on neutrophil infiltration**.** Eyes of C57BL6/J mice were infected with *S. aureus* strains LAC, LAC ΔΔΔΔΔ, LAC Δ*leukocidin*, or LAC Δ*hla*. Eyes were harvested at 12, 24, or 36 h post-infection and analyzed by flow cytometry. (**A**) Representative gating strategy used to identify neutrophils. (**B**) Quantitation of the total number of CD45^+^ CD11b^+^ myeloid-derived cells, (**C**) Ly6G^+^ Ly6C^+^ neutrophils, and (**D**) ADAM10^+^ neutrophils. Eyes infected with LAC Δ*hla* exhibited statistically less infiltrating CD45^+^ CD11b^+^ Ly6G^+^ Ly6C^+^ ADAM10^+^ neutrophils compared to eyes infected with LAC at 24 h post-infection. (**E**) Percentage of neutrophils that were ADAM10^+^. No differences in the percent of ADAM10^+^ neutrophils were observed between LAC or any of the isogenic toxin-deficient mutants at any time point. Data represent the mean number of cells or percentage ± SEM of *n* = 6 eyes/group. Statistical significance is indicated by *P < 0.05; **P < 0.01; ***P < 0.001 (Kruskal-Wallis test with Dunn’s multiple correction test).

To determine whether the absence of PFTs affected neutrophil infiltration during endophthalmitis, we performed flow cytometry to quantify infiltrating neutrophils in eyes infected with LAC, LAC ΔΔΔΔΔ, LAC Δ*leukocidin*, or LAC Δ*hla* at 12, 24, or 36 h post-infection. We focused on neutrophil infiltration as neutrophils are the primary immune cells that infiltrate into the eye during *S. aureus* endophthalmitis (30, 33). Figure 8A summarizes the flow cytometry gating strategy. After exclusion of doublets, live cells were gated on CD45^+^CD11b^+^ to identify myeloid cells. Neutrophils were defined as Ly6G^+^Ly6C^+^ cells, and cell surface expression of ADAM10, the receptor for the alpha toxin, was analyzed within the neutrophil population.

Figure 8B shows the quantity of CD45^+^CD11b^+^ myeloid cells in infected eyes. At 12 h postinfection, there were no differences in the number of infiltrating myeloid cells in eyes infected with LAC, LAC ΔΔΔΔΔ (*P* = 0.3074), LAC Δ*leukocidin* (*P* = 0.4349), or LAC Δ*hla* (*P* = 0.5743). Similarly, at 24 h post-infection, there were no differences in the number of myeloid cells in eyes infected with LAC or LAC ΔΔΔΔΔ (*P* > 0.9999) or LAC Δ*leukocidin* (*P* = 0.2375). However, there was a greater number of infiltrating myeloid cells in eyes infected with LAC compared to eyes infected with LAC Δ*hla* (*P* = 0.0341) at 24 h post-infection. At 36 h, we observed less infiltrating myeloid cells in eyes infected with LAC ΔΔΔΔΔ (*P* = 0.4591) or LAC Δ*hla* (*P* = 0.4249) compared to eyes infected with LAC, but these differences were not statistically significant. No differences were observed in eyes infected with LAC or LAC Δ*leukocidin* (*P* > 0.9999) at 36 h post-infection.

Figure 8C shows the quantity of neutrophils in infected eyes. At 12 h post-infection, there were no differences in the number of infiltrating neutrophils in eyes infected with LAC, LAC ΔΔΔΔΔ (*P* = 0.8655), LAC Δ*leukocidin* (*P* = 0.7590), or LAC Δ*hla* (*P* = 0.4954). By 24 h post-infection, there were similar numbers of neutrophils in eyes infected with LAC, LAC ΔΔΔΔΔ (*P* > 0.9999), or LAC Δ*leukocidin* (*P* =0.4249). There were greater numbers of infiltrating neutrophils in eyes infected with LAC compared to eyes infected with LAC Δ*hla* (*P* = 0.0239) at 24 h post-infection.

At 36 h, we observed fewer infiltrating neutrophils in eyes infected with LAC ΔΔΔΔΔ (*P* = 0.3074) or LAC Δ*hla* (*P* = 0.2825) compared to eyes infected with LAC, but these differences were not statistically significant. No differences in infiltrating neutrophils were observed in eyes infected with LAC or LAC Δ*leukocidin* (*P* > 0.9999) at 36 h post-infection.

Figure 8D through E depicts the number and percent of ADAM10^+^ neutrophils, respectively. At 12 h post-infection, there was no difference in the number of ADAM10^+^ neutrophils in eyes infected with LAC, LAC ΔΔΔΔΔ (*P* > 0.9999), LAC Δ*leukocidin* (*P* > 0.9999), or LAC Δ*hla* (*P* > 0.9999). This resulted in a similar percentage of ADAM10^+^ neutrophils in LAC, LAC ΔΔΔΔΔ (*P* = 0.4767), LAC Δ*leukocidin* (*P* = 0.8107), or LAC Δ*hla* (*P* > 0.9999). At 24 h post-infection, there were similar numbers of ADAM10^+^ neutrophils in eyes infected with LAC, LAC ΔΔΔΔΔ (*P* > 0.9999), or LAC Δ*leukocidin* (*P* = 0.7490). This resulted in a similar percentage of ADAM10^+^ neutrophils in eyes infected with LAC, LAC ΔΔΔΔΔ (*P* > 0.9999), or LAC Δ*leukocidin* (*P* > 0.9999). Compared to eyes infected with LAC, eyes infected with LAC Δ*hla* had fewer ADAM10^+^ neutrophils (*P* = 0.0429); however, this difference was likely due to the fact that there were fewer infiltrating neutrophils in eyes infected with LAC Δ*hla*. There was no difference in the percentage of ADAM10^+^ neutrophils between eyes infected with LAC or LAC *Δhla* (*P* > 0.9999). At 36 h post-infection, there was no difference in the number of ADAM10^+^ neutrophils in eyes infected with LAC, LAC ΔΔΔΔΔ (*P* > 0.7590), LAC Δ*leukocidin* (*P* > 0.9999), or LAC Δ*hla* (*P* > 0.4591). This resulted in a similar percentage of ADAM10^+^ neutrophils in eyes infected with LAC, LAC ΔΔΔΔΔ (*P* > 0.9999), LAC Δ*leukocidin* (*P* > 0.9999), or LAC Δ*hla* (*P* > 0.9999). On average, 40%–60% of neutrophils were ADAM10,^+^ and this percentage varied depending on the time point analyzed. Collectively, these results demonstrated that in the absence of the alpha toxin, fewer neutrophils infiltrated into the eye during *S. aureus* endophthalmitis. Further, we showed that a subpopulation of infiltrating neutrophils had cell surface expression of ADAM10^+^, suggesting that these cells may be potential alpha toxin targets.

### Intraocular alpha toxin during infection

**Fig 9:**
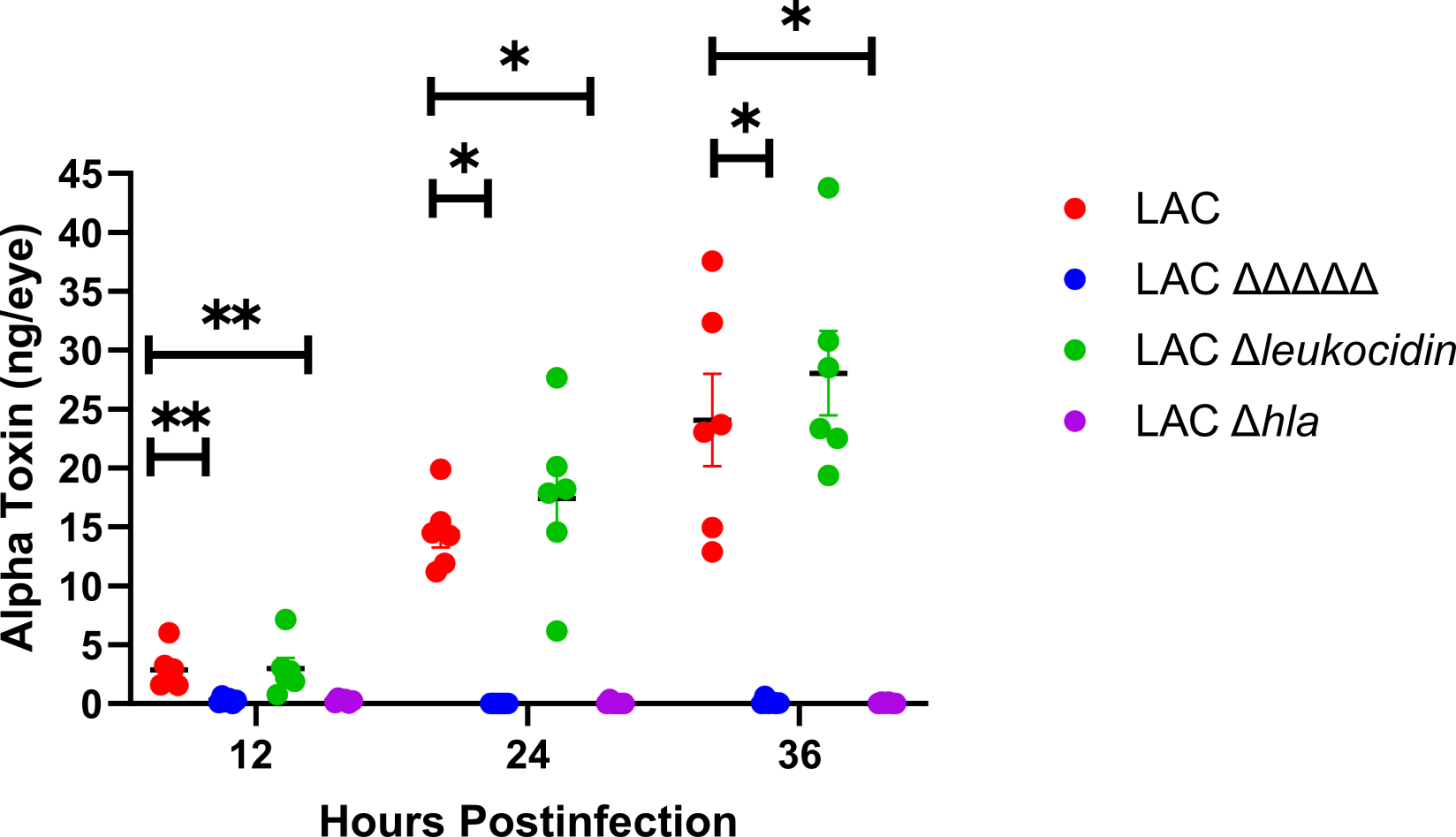
Intraocular alpha toxin during infection**.** Eyes of C57BL6/J mice were infected with *S. aureus* strains LAC, LAC ΔΔΔΔΔ, LAC Δ*leukocidin*, or LAC Δ*hla*. Eyes were harvested and homogenized at 12, 24, or 36 h post-infection. Alpha toxin was detected in eye homogenates using sandwich ELISA. Alpha toxin was detected in eyes infected with LAC or LAC Δ*leukocidin* at 12, 24, or 36 h post-infection. No differences in alpha toxin were observed between eyes infected with LAC or LAC Δ*leukocidin* at any time point. Eyes infected with LAC ΔΔΔΔΔ or LAC Δ*hla* produced little to no alpha toxin at 12, 24, or 36 h post-infection, and the average alpha toxin produced in these eyes was below the limit of detection. Data represent mean ng/eye ± SEM of *n* = 6 eyes/group. Statistical significance is indicated by *P < 0.05; **P < 0.01; ***P < 0.001 (Kruskal-Wallis test with Dunn’s multiple correction test).

Figure 9 depicts quantitation of intraocular alpha toxin at 12, 24, and 36 h post-infection. Alpha toxin was detected in increasing quantities at 12, 24, and 36 hours postinfection in eyes infected with LAC or LAC Δ*leukocidin*. There were no statistical differences in intraocular alpha toxin in eyes infected with LAC or LAC Δ*leukocidin* at any time point (*P* > 0.9999 at all time points). As expected, no alpha toxin was detected in eyes infected with LAC ΔΔΔΔΔ or LAC Δ*hla*. These results confirm that alpha toxin is being produced in the eye during *S. aureus* endophthalmitis. Taken together, these results suggest that the differences in infection outcomes in eyes infected with mutants deficient in alpha toxin can likely be attributed to the loss of that toxin alone, making alpha toxin an important driver of pathogenesis in *S. aureus* endophthalmitis.

## DISCUSSION

Bacterial endophthalmitis is a medical emergency that requires immediate medical attention to prevent permanent damage to intraocular tissue and irreversible vision loss. Endophthalmitis cases caused by *S. aureus* frequently result in poor visual outcomes due in part to the bacteria’s many virulence factors. Successful treatment of *S. aureus* endophthalmitis is further complicated by the increasing prevalence of multidrug-resistant strains (34, 35). Even if a strain is susceptible to the antibiotics used to treat endophthalmitis, secreted toxins can continue to drive ocular tissue damage and inflammation. Experimental endophthalmitis caused by a *S. aureus agr-*deficient mutant resulted in attenuated infections, suggesting that toxin production contributes to the severity of *S. aureus* endophthalmitis (16, 17). Similarly, alpha toxin and leukotoxins have been implicated in the pathogenesis of ocular *S. aureus* infections (5, 36–38). In the current study, we assessed the role of *S. aureus* PFTs, which are all under the control of Agr, in the pathogenesis of experimental endophthalmitis.

We first assessed whether PFTs were expressed in an *ex vivo* environment, using explanted rabbit vitreous and BHI as growth media (Fig. 2). We previously tested whether *S. aureus* strain JE2, a derivative of USA300 LAC, expressed leukocidins *ex vivo* and reported a similar reduction in relative expression of all leukocidins, except for *lukED* (30). Here, we also report that *hla* expression was less in vitreous relative to expression in BHI. Leukocidins have been reported to have lytic effects on host cells *in vitro* in concentrations as low as 1 nM, suggesting that even low-level expression may result in biological activity in the ocular environment (39, 40). Several studies have shown that leukocidin expression is dependent on the growth media, suggesting that environmental factors influence the expression of these toxins (41–43). An additional factor that may influence the expression of these toxins is nutrient availability. BHI is a nutrient-rich medium derived from mammalian tissue. On the other hand, vitreous consists of ~98% water, collagen, hyaluronan, trace amounts of salt, and a glucose concentration (3.5 mM) lower than that of blood glucose in nondiabetic individuals (44, 45). During intraocular infections, the presence of infiltrating cells and the inflammatory milieu may provide nutrients, influencing PFT expression.

The absence of PFTs in *S. aureus* during endophthalmitis resulted in a greater percent retention in the A-wave response and a trend toward greater percent retention in the B-wave response (Fig. 5). Notably, the absence of alpha toxin alone resulted in greater percent retention in both the A-wave and B-wave responses. This is consistent with findings in the experimental rabbit endophthalmitis model, where infections with a strain deficient in alpha toxin resulted in a significantly attenuated B-wave response. In that study, infection with a strain deficient in alpha toxin, beta toxin, and gamma toxin resulted in retinal function greater than that of infection with wild-type or single-toxin mutants (5). PVL has been shown to activate Müller cells, a cellular driver of the B-wave response, in explanted rabbit retinas (26, 27, 46). In the current study, the retained B-wave responses of eyes infected with a strain deficient in all leukocidins was similar to that of eyes infected with the wild-type strain. Given how leukocidins have limited tropism to murine leukocytes, PVL may also have limited tropism toward murine Müller cells. As a result, even if Müller cells have cell surface expression of C5aR, the reduced tropism toward this receptor may explain why there was no difference in retinal function when eyes were infected with a leukotoxin-deficient mutant (47, 48). Although PVL colocalization with C5aR has been observed in retinal ganglion cells and displaced amacrine cells, colocalization of PVL with Müller cells via C5aR was not observed (26, 27). However, since purified PVL was injected into the rabbit eye, the concentrations tested may have been different than what is produced *in vivo* during infection.

When eyes were infected with mutants deficient in all PFTs or in alpha toxin alone, we observed a reduction in the intraocular *S. aureus* burden (Fig. 6). This difference was attributed to the absence of alpha toxin as the absence of all leukocidins did not recapitulate the reduction in intraocular burden. A reduction in bacterial burden has been observed in a scratch model of *S. aureus* keratitis when both young and old mice were infected with an alpha toxin-deficient mutant (49). However, intrastromal injections of an alpha toxin-deficient mutant in a rabbit model of *S. aureus* keratitis resulted in similar bacterial burdens, but reduced corneal pathology (38, 50). Additional studies have shown that infections with alpha toxin-deficient mutants had reduced bacterial burden in other tissues, such as the brain, lung, and skin (51–54). Collectively, reports of reduced bacterial burden in other tissue types reinforce our findings that in the absence of alpha toxin, *S. aureus* proliferation in intraocular infections is limited.

Damage to ocular tissue is driven by not only bacterial virulence but also by the host inflammatory response to infection. We showed with slit-lamp imaging (Fig. 3), histology (Fig. 4), MPO ELISA, Bio-Plex analysis of cytokines and chemokines (Fig. 7), and flow cytometry (Fig. 8) a trend in reduced inflammation in eyes infected with the LAC ΔΔΔΔΔ or LAC Δ*hla* mutants. We observed statistically less intraocular IL-1α, IL-6, and G-CSF in eyes infected with LAC Δ*hla* compared to eyes infected with LAC. Further, we observed a decrease in intraocular MPO and IL-1β, but not G-CSF, in eyes infected with LAC ΔΔΔΔΔ compared to eyes infected with LAC. Collectively, eyes infected with the PFT-null mutant had reduced intraocular inflammation, and a similar reduction was observed in eyes infected with an alpha-toxin deficient mutant. Eyes infected with the leukocidin-null mutant did not have significantly reduced intraocular burden, suggesting that alpha toxin is a main contributor to intraocular infections. Several studies have shown that IL-1α, IL-1β, and TNF-α contribute to the host response to *S. aureus* infections (55–58). PVL, gamma toxin, LukGH, and alpha toxin have been shown to activate the NOD-, LRR- and pyrin domain-containing 3 (NLRP3) inflammasome *in vitro* in human and murine monocytes and macrophages to induce the production of IL-1β (59–62). In a murine model of *S. aureus* endophthalmitis, IL-1β has been shown to be essential in regulating the immune response to *S. aureus* as the absence of the NLRP3 inflammasome in mice resulted in a greater bacterial burden (58). Furthermore, it has been shown that neutrophils are the main cells that produce IL-1β during corneal *S. aureus* infections, and this production is driven by alpha toxin (63). While there were no statistical differences in intraocular TNF-α among the infection groups in our study, we observed a decrease in the quantity of TNF-α in eyes infected with the LAC ΔΔΔΔΔ mutant. In experimental *Bacillus* endophthalmitis, TNF-α knockout mice exhibited a decrease in neutrophil infiltration, but this facilitated greater bacterial replication (64). While no studies to date have investigated the role of TNF-α in *S. aureus* endophthalmitis, given this cytokine’s protective role, a deficiency in TNF-α may also facilitate *S. aureus* replication. IL-6 has been reported to have an immunomodulatory effect in *S. aureus* corneal infection models, where this cytokine may prime neutrophils to facilitate bacterial clearance (65). While no studies have investigated the role of G-CSF in ocular infections, G-CSF modulates neutrophil function and has been shown to augment the resolution of *S. aureus* wound infections (66, 67). No differences in intraocular KC or MIP-2 were observed among eyes infected with LAC or the three mutants tested in the current study. We reported that in *S. aureus* endophthalmitis, the absence of CXCL-1 (KC) limits intraocular inflammation early in infection but does not affect bacterial burden. In that study, the absence of CXCL-2 (MIP-2) in mice did not affect intraocular inflammation or bacterial burden (68).

Injection of purified alpha toxin into mouse eyes was reported to induce dose-dependent inflammation and the production of IL-1β, TNF-α, and KC, but not MIP-2 or IL-6 (24). In the current study, alpha toxin concentrations detected at 12 h post-infection were below the lowest dose tested (0.01 μg), while concentrations detected at 24 and 36 h were between the lowest (0.01 μg) and intermediate (0.1 μg) concentrations tested (24). In the current study, ocular pathology, neutrophil influx, and proinflammatory mediator synthesis paralleled the increasing CFU and amounts of alpha toxin within the eye. In the study mentioned previously (24), significant increases in inflammatory mediators were detected only when 1 μg of alpha toxin was injected into the mouse eye (24). This concentration of alpha toxin may not have been physiologically relevant since in the current study, intraocular alpha toxin concentrations only reached an average of 30 ng or less per eye during infection. While it is known that other *S. aureus* components contribute to the inflammatory response observed during endophthalmitis and the general decrease in inflammation might also be attributed to the reduced bacterial burden in eyes infected with LAC ΔΔΔΔΔ or LAC Δ*hla*, the alpha toxin has been shown to synergistically enhance the inflammatory response caused by bacterial cell wall components in a murine peritoneal infection model (69). This raises the possibility that alpha toxin may also augment the inflammatory response during intraocular infections.

ADAM10 is the cellular receptor for the alpha toxin and is required for toxin-mediated pore formation in host cells. Our findings demonstrate that 40%–60% of infiltrating neutrophils were ADAM10^+^ (Fig. 8), identifying this subpopulation as potential targets during *S. aureus* endophthalmitis. Other studies have reported that neutrophils have cell surface expression of ADAM10, although the cell surface expression is less compared to that of other cell types (63, 70, 71). Some studies suggest that in inflammatory environments, neutrophils upregulate the expression of ADAM10 (63, 72). In the current study, we detected the presence of alpha toxin in infected eyes as early as 12 h post-infection, with alpha toxin concentrations increasing during the course of infection (Fig. 9). Prior to the identification of ADAM10 as the receptor for the alpha toxin, studies reported that alpha toxin could bind to granulocytes, but the heptameric structure failed to insert into the membrane (73). The concentration required to induce neutrophil lysis is reported to be greater than 50 μg/mL (74, 75). While the concentration in infected eyes in our study was substantially less, alpha toxin has been shown to have sublytic effects on neutrophils at microgram concentrations (75). Further, in a sepsis model, alpha toxin has been shown to augment the inflammatory response through platelet-neutrophil aggregation. Deletion of ADAM10 in both myeloid cells and platelets was protective in that model (76). Pharmacological inhibition of ADAM10 led to a reduction in IL-1β release from neutrophils during mouse corneal infection (63). Further, alpha toxin has been shown to suppress neutrophil chemotaxis and calcium signaling *in vitro* and *in vivo*, leading to impaired recruitment of neutrophils in an ear wound infection model (75). Collectively, these findings suggest that while the alpha toxin may not reach a concentration that leads to neutrophil lysis in the eye, this toxin may contribute to the inflammatory response and impair bacterial clearance during *S. aureus* endophthalmitis.

In the current study, we detected ADAM10 surface expression on a subpopulation of neutrophils, but not on all neutrophils. However, there may be other cell types that express ADAM10 on their cell surface. ADAM10 has been shown to be essential for retinal development in chicken and mouse models (77, 78). In 3-month-old mice, ADAM10 expression was detected in retinal ganglion cells, the inner nuclear layer, and outer limiting membrane, suggesting that multiple cell types in the retina may be potential alpha toxin targets (79). ADAM10 has been detected in the vitreous proteome of adult diabetic retinopathy patients, implicating that ADAM10 can have pathological effects in ocular diseases (80). Additionally, endothelial cells are known to have cell surface expression of ADAM10, and the same holds true for retinal endothelial cells (81). ADAM10 has been shown to be upregulated in immortalized human retinal pigment epithelium cells infected with Epstein-Barr virus (82). The expression of ADAM10 across diverse ocular cell types raises the possibility that the alpha toxin may interact with multiple cell types within the eye. The improved A-wave and B-wave responses in infections with mutants deficient in alpha toxin suggest that the alpha toxin might negatively interact with or cause downstream changes in cells responsible for retinal function, resulting in dysfunction. Future studies will address whether alpha toxin targets retinal cells through ADAM10 interactions during infections.

We report here, for the first time, that alpha toxin is detected in the intraocular environment during *S. aureus* endophthalmitis (Fig. 9). Alpha toxin was detected at 12 h post-infection, and concentrations increased as the infection progressed. Expression of several *S. aureus* virulence genes is regulated by Agr quorum sensing, which upregulates the expression of secreted virulence factors as *S. aureus* population density increases (15). Given that we detected alpha toxin in the eye as early as 12 h post-infection, this suggests that *S. aureus* reaches a quorum at or around 12 h post-infection in this model. In eyes infected with LAC or LAC Δ*leukocidin,* we observed an increase from 10^6^ CFU/eye to 10^7^ CFU/eye from 12 to 24 h post-infection (Fig. 6), as well as an increase in alpha toxin (Fig. 9). We observed a further increase in alpha toxin at 36 h post-infection, but intraocular CFU remained at 10^7^ CFU/eye. The continued increase in alpha toxin at 36 h post-infection without an increase in intraocular burden may reflect continued positive regulation by Agr. Agr signaling is positively autoregulated as activation of AgrA promotes transcription from the P2 promoter, leading to increased *agrBCDA* expression and amplification of quorum sensing signaling (15). Amplification of Agr *in vivo* would result in greater alpha toxin in the intraocular environment, which contributes to damage to the ocular tissue and intraocular inflammation.

The presence of alpha toxin in the eye during infection suggests that this toxin would be a viable target to mitigate the severity of *S. aureus* endophthalmitis. Immunization with an alpha toxin toxoid was protective in a rabbit model of *S. aureus* keratitis. Although immunization did not decrease bacterial burden, infected corneas exhibited smaller epithelial erosions (37, 83). A second study also reported reduced corneal damage in a *S. aureus* keratitis model when rabbits were treated with a high-affinity monoclonal antibody against alpha toxin (84). These studies suggested that targeting alpha toxin might be an effective strategy to treat ocular infections. To therapeutically neutralize PFTs, Coburn et al. (85) tested the efficacy of rabbit nanosponges in a murine model of *S. aureus* endophthalmitis. Nanosponges are nanoparticles coated with erythrocyte membranes that can sequester PFTs, thereby limiting the interactions of PFTs with host cell membranes and reducing cytotoxicity (86). Although treating *S. aureus-*infected eyes with nanosponges alone did not result in improved retinal function, treatment with gatifloxacin and nanosponges resulted in improved retinal function and reduced bacterial burden compared to that of eyes treated with gatifloxacin alone (85). Taken together, neutralizing alpha toxin should reduce toxin-mediated damage during ocular infections; however, neutralization alone may be insufficient to improve infection outcomes without concurrent antimicrobial and anti-inflammatory therapy.

We reported no direct role for leukocidins in the pathogenesis of experimental *S. aureus* endophthalmitis. However, a limitation of this study might be the use of a murine model. Leukocidins, except for LukED and HlgAB, have limited tropism toward murine leukocytes (48, 87, 88). Despite LukED having a tropism for murine leukocytes, we reported that LukED did not contribute to the pathogenesis of *S. aureus* endophthalmitis in mice despite observing expression of *lukED* in explanted vitreous and *in vivo* during infection (30). In rabbit models of endophthalmitis, gamma toxin did not contribute to intraocular inflammation or retinal function loss (5, 29). However, although studies have reported no differences in virulence of strains deficient in single leukocidins, cytotoxic activities may have been due to synergistic activities during infection. For example, synergism between LukGH and PVL has been reported, where sublytic concentration of PVL upregulated CD11b surface expression. Given that CD11b is the receptor for LukGH, upregulation of CD11b may enhance LukGH cytoxicity (89). This upregulation in CD11b may disrupt intracellular killing, facilitating *S. aureus* persistence after phagocytosis by macrophages and neutrophils (42). In addition, recombinant PVL has been found to synergize with recombinant gamma toxin to amplify the release of IL-1β *in vitro* in human alveolar macrophages (90). In ocular infection, the amplification of IL-1β might result in an increase in inflammatory-mediated ocular tissue damage. However, although some studies suggest synergism among leukocidins, a different study demonstrated that LukF-PV can bind with LukE, which impedes cytotoxic activity in primary murine bone marrow cells (91). In that study, strains that produced both PVL and LukED were less virulent than strains only producing LukED (91). The antagonistic relationship between PVL and LukED was further supported by findings showing that uncoupling these antagonistic effects promoted *S. aureus* lung colonization (91). Given that studies have reported synergism between leukocidins, infections with the leukocidin-null mutant in a rabbit model of endophthalmitis may reveal potential synergism of leukocidins in the eye.

Synergistic effects have also been reported between leukocidins and the alpha toxin. In a model of septic arthritis, the presence of both alpha toxin and gamma toxin correlated with increased disease severity (92). LukGH and the alpha toxin have also been found to have synergistic effects in a mouse model of orthopedic implant infection, where *S. aureus* in biofilms secreted both toxins, inducing macrophage dysfunction (93). In that model, infections with a mutant deficient in both LukGH and alpha toxin resulted in a decrease in bacterial burden and increased macrophage infiltration compared to infection with single-toxin mutants (93). While our results suggest that alpha toxin is a key player during murine *S. aureus* endophthalmitis, potential alpha toxin-leukocidin interactions may not have occurred due to the limited tropism of leukocidin toward murine neutrophils.

In conclusion, we report here that the absence of *S. aureus* PFTs results in a reduced bacterial burden, preservation of retinal function, and decrease in intraocular inflammation in experimental endophthalmitis. Notably, the absence of alpha toxin alone resulted in similar attenuation of infection outcomes, while the absence of leukocidins did not significantly attenuate infection outcomes. Collectively, these results suggest alpha toxin plays a dominant role in shaping disease severity in the murine model of *S. aureus* endophthalmitis. The absence of leukocidins and the presence of alpha toxin have been screened in clinical ocular isolates, and *hla* has been present in almost every isolate (2, 94–96). In the current study, we utilized an isogenic family of strains in the USA300 background. However, similar analysis of infections with *S. aureus* isolates in other backgrounds could strengthen the hypothesis that alpha toxin is a dominant player in shaping disease severity in experimental models of endophthalmitis. Future studies could also investigate the efficacy of anti-alpha toxin or alpha toxin inhibitors with antibiotics for ocular infections caused by clinical *S. aureus* isolates. Also of interest is the role of ADAM10 in inflammation during ocular infections. The clinical goal is to rapidly limit bacterial proliferation, neutralize damaging toxins, and limit intraocular inflammation, thereby protecting vision in patients with this disease.

## MATERIALS AND METHODS

### Bacteria

The strains used in this study are listed in Table 1. We used *S. aureus* strain USA300 LAC, LAC ΔΔΔΔΔ, LAC Δ*leukocidin*, and LAC Δ*hla* (31). These strains were a kind gift from Dr. Victor Torres (St. Jude Children’s Research Hospital, Memphis, TN). USA300 LAC was originally isolated from a soft skin and tissue infection from an inmate in the Los Angeles County Jail (97).

### Bacterial growth *in vitro*

To generate growth curves, bacteria were cultured in brain heart infusion (BHI; VWR, Radnor, PA) at 37°C with aeration at 220 RPM for 18 h. After pelleting at 4,150 × *g* for 5 min, the supernatant was discarded, and the pellet was washed with phosphate-buffered saline (PBS, pH 7.4). The washed pellet was then resuspended and diluted to ~ 10^3^ colony-forming units (CFU)/mL in either fresh BHI or in explanted rabbit vitreous (Pelfreez Biologics, Rogers, AR) and incubated at 37°C. Every 2 h for 18 h, 20 µL aliquots were serially diluted 10-fold in PBS and then plated onto BHI agar plates. Plates were incubated at 37°C for 18 h, and colonies were counted to generate a growth curve. Growth rates were calculated during the exponential phase using the following equation, *N_t_* = *N*_0_ × (1 + *r*)*^t^*, where *N*_t_ is the bacterial concentration at the end of the exponential phase, *N*_0_ is the bacterial concentration at the start of the exponential phase, *r* is the growth rate (hour^−1^), and *t* is the time passed (30, 98).

### Bacterial RNA isolation and quantitative PCR

Bacterial RNA was isolated from wild-type LAC cultures grown in BHI or explanted vitreous for 18 h at 37°C, as described previously (30, 98). Overnight cultures were centrifuged at 4,150 × *g* for 5 min, the supernatant was discarded, and the pellet was resuspended with RLT lysis buffer (RNeasy Minikit; Qiagen, Germantown, MD). The resuspended pellet was transferred into a tube containing 0.1 mm beads (Biospec Products Inc, Bartesville, OK) and then homogenized with a mini bead beater (Biospec Products Inc.) at 5,000 RPM for 60 s. Samples were then briefly pulsed, transferred to a snap cap tube, and then centrifuged for 2 min at 16,000 × *g*. RNA was isolated using the RNeasy kit (Qiagen), treated with DNase I (Zymo Research, Irvine, CA), and purified with the RNA Clean and Concentrator-5 (Zymo Research), all according to the manufacturer’s instructions. After purification, the RNA concentration and purity were determined using NanoDrop (Thermo Fisher Scientific, Waltham, MA). RT-qPCR (Applied BioSystems 7500; Thermo Fisher Scientific) was performed to determine the expression of PFT genes (*hla*, *lukS-PV*, *lukF-PV*, *lukE*, *lukD*, *lukG*, *lukH*, *hlgA*, *hlgB*, and *hlgC*) using the iTaq Universal SYBR Green One-Step kit (Bio-rad, Hercules, CA). 16s rRNA was used as the housekeeping control. Relative expression was calculated using the ΔC_T_ method. Table 2 summarizes the primers used for this experiment.

**TABLE 2:** Primers used in this study

Gene		Sequence (5'−3')	Reference
*lukS-PV*	Fwd	CAT CCA TAT TTC TGC CAT ACG TT	(30)
	Rev	CAC AGT GGT TTC AAT CCT TCA T	(30)
*lukF-PV*	Fwd	CGG AAT CTG ATG TTG CAG TTG T	(30)
	Rev	TCC AAT ACA GTT GAT GCA GCT C	(30)
*lukE*	Fwd	CAC CTT TAG CAT CTC CGA TTC A	(30)
	Rev	CTA CTT ACA TCC TCC GTA CGT TTG	(30)
*lukD*	Fwd	GGC AGC CGG AAA CAT TAA TTC	(30)
	Rev	ACT AAC CGA AAC ATT ATA CTT ACC G	(30)
*lukG*	Fwd	AAT ATG GGA CAT GAC CAT ACG AG	(30)
	Rev	TCG CCC ATA AAT TTC CAT TTC G	(30)
*lukH*	Fwd	CTT GAA GTA TGG TGG AGA AGT GA	(30)
	Rev	AGC AAA GCT TAG TTC AGG GT	(30)
*hlgA*	Fwd	CGA GAC TAT TTC GTC CCA GAT AA	(30)
	Rev	CAC CTT TAC CTC TTT CGT GTG A	(30)
*hlgB*	Fwd	TAA GCG TACTAT CAC ACA GAC AAG	(30)
	Rev	AAG CCA TTC CAA CGA ATT TGG	(30)
*hlgC*	Fwd	CCC TTC ATT TAT CGC CAC AGT A	(30)
	Rev	AAT FFC ATG AGT GAC ATC CA	(30)
*hla*	Fwd	ATT CTT GGA ACC CGG TAT ATG G	This study
	Rev	GAA CTT GCT TTG TTA GGA TCA AGG	This study
*16s rRNA*	Fwd	GTG GAG GGT CAT TGG TGG AAA CT	(30)
	Rev	CAC TGG TGT TCC TCC ATA TCT C	(30)

### Mice

Male C57BL/6 mice were purchased from Jackson Labs (stock number 000664; Bar Harbor, ME). All *in vivo* experiments followed the guidelines and recommendations of the University of Oklahoma Health Sciences Center Institutional Animal Care and Use Committee (protocol 24-038), the ARVO Statement for the Use of Animals in Ophthalmic and Vision Research, and the Guide for the Care and Use of Laboratory Animals. Mice were housed in biosafety level 2 conditions on a 12-h dark/light cycle for 2 weeks to equilibrate their microbiota. *In vivo* experiments were performed on 8- to 10-week-old male mice.

### Murine endophthalmitis model

USA300 LAC and all isogenic mutants were cultured in BHI at 37°C for 18 h. Overnight cultures were then diluted to approximately 10,000 CFU/µL (1 × 10^7^ CFU/mL) prior to infection. Mice were anesthetized with isoflurane before receiving an intravitreal injection in the mid-vitreous of the right eye with 5,000 CFU in 0.5 µL using a sterile beveled capillary needle (Kimble Glass Company, Vineland, NJ). The contralateral left eye served as an uninfected control (99). Only male mice were used as sex is not a biological factor in murine endophthalmitis (100–102).

### Slit-lamp examination

Ocular infections were monitored by slit-lamp examination at 12, 24, or 36 h post-infection (103, 104). Mice were anesthetized with isoflurane, followed by administration of topical drops for anesthesia (0.5% proparacaine HCl; Alcon Laboratories, Fort Worth, TX), and pupil dilation (10% topical phenylephrine hydrochloride; Paragon BioTeck, Portland, OR). Images were captured with the Micron IV slit-lamp imaging system (Phoenix Research Labs Inc., CA).

### Histology

Ocular pathology was assessed by histology (30, 101, 105). At 12, 24, or 36 h post-infection, mice were euthanized by euthanized by CO_2_ inhalation, and eyes were harvested and incubated in Perfix fixative (Exalibur Pathology, Moore, OK) overnight at room temperature. Eyes were then transferred into 70% ethanol prior to paraffin embedding. Sections were stained with hematoxylin and eosin and then scanned at 40× using the Motic EasyScan scanner (Motic Instruments Inc., Richmond BC).

### Electroretinography

Retinal function was analyzed by electroretinography (ERG) to quantify A-wave and B-wave responses, as described previously (30, 106, 107). The A-wave measures the function of rod photoreceptors, and the B-wave measures the function of bipolar neurons and Müller glia cells (108). Mice were dark-adapted for a minimum of 6 h prior to ERG analysis. ERGs were performed with the Espion E2 system (Diagnosys LLC, Lowell, MA) at 12, 24, or 36 h post-infection. Mice were anesthetized with an intraperitoneal injection of ketamine and xylazine (ketamine HCl, 85 mg/kg; Covetrus, Portland, ME; xylazine, 14 mg/kg; AnaSed, Akorn, Decatur, IL). Topical drops for anesthesia and dilation were administered as described above. Gold-wire electrodes were positioned on the corneal surface of each eye, while reference and ground electrodes were placed on the forehead and tail, respectively. Both eyes were stimulated simultaneously with five sequential flashes at an intensity of 1,200 cd·s/m², and A- and B-wave amplitudes were recorded. Retinal function was expressed as percent retention of A- and B-wave amplitudes relative to the uninfected contralateral eye using the following calculation: 100 − {(1 − [Experimental A-wave or Experimental B-wave amplitude/Control A-wave or Control B-wave amplitude])} ×100, respectively.

### Intraocular bacteria quantitation

At 12, 24, or 36 h post-infection, mice were euthanized by CO_2_ inhalation. After euthanasia, eyes were harvested and transferred into a sterile bead beater tube containing sterile 1 mm glass beads (BioSpec Products, Inc.) and 400 µL PBS. Eyes were then homogenized twice in a mini-bead beater (BioSpec Products, Inc) for 60 s at 5,000 RPM. Eye homogenates were diluted 10-fold, plated on BHI agar plates, incubated overnight at 37°C, and bacterial colonies were counted, as described previously (30, 105, 107).

### Myeloperoxidase

Myeloperoxidase (MPO) was quantified at 12, 24, or 36 h post-infection, as described previously (30, 105, 107). Mice were euthanized as described above, and eyes were harvested and placed in PBS containing cOmplete EDTA-free Protease Inhibitor (Roche Diagnostics, Indianapolis, IN) and homogenized as described above. Intraocular MPO was determined using sandwich ELISA following the kit manufacturer’s instructions (Hycult Biotech, Plymouth Meeting, PA). The lower limit of detection of this kit is 2 ng/mL.

### Cytokine and chemokine quantification

The following cytokines/chemokines were analyzed: IL-1α, IL-1β, IL-6, IL-10, TNF-α, G-CSF, KC, and MIP-2 using a custom Milliplex Mouse Cytokine/Chemokine magnetic bead kit (Sigma Aldrich, Darmstadt, Germany). After an initial wash step, 25 µL of the standards, control, or samples and 25 µL of assay buffer were added to each well of a 96-well plate. Twenty-five microliters of the mixed magnetic beads was added to each well, and then the plate was sealed and incubated at 4°C in a plate shaker for 18 h. Following incubation, the plate was washed twice, covered in foil, and then incubated with 25 µL of the detection antibody at room temperature for 2 h on a plate shaker. The plate was washed twice, then incubated with 25 µL of streptavidin-PE at room temperature, covered in foil, and incubated for 30 min on a plate shaker. The plate was washed twice and then resuspended with sheath fluid. The beads were then resuspended by agitation on a plate shaker for 5 min. Samples were analyzed on the Bio-Plex 200 (Biorad). The lower limit of detection for this assay is 3.2 pg/mL.

### Flow cytometry

Flow cytometry analysis was performed as described previously (30, 105). At 12, 24, or 36 h post-infection, mice were anesthetized with ketamine and xylazine as described above and then perfused with PBS. Following euthanasia, eyes were harvested and stored in 2 mL of Roswell Park Memorial Institute (RPMI) 1640 media without L-glutamine (Gibco, Grand Island, NY, USA) and supplemented with 10% fetal bovine serum (Gibco) until further processing. Eyes were mechanically dissociated by gently pressing the tissue through a 70-µm cell strainer (Thermo Fisher Scientific, Waltham, MA, USA) using the wide end of a sterile 1-mL syringe plunger. Following dissociation, eyes were enzymatically digested with 2 mg/mL type I collagen (Worthington, Lakewood, NJ) for 15 min at 37°C with gentle agitation. The remaining tissue was mechanically dissociated by gently pressing against the cell strainer to create a single-cell suspension. Cells were then transferred to a 96-well plate, centrifuged at 300 × *g* for 5 min, and the supernatant was discarded. Cells were then stained for viability with LiveDead Aqua (Thermo Fisher Scientific) at 4°C for 20 min. Cells were washed with staining buffer and then stained with the following antibodies, all from Biolegend, unless otherwise noted: anti-CD45 AF700 (clone 30-F11), Ly6G APC (clone 1A8), Ly6C Alexa Fluor 488 (clone HK1.4), Cd11b BV421 (clone M1/70), and ADAM10 PE (clone 139712, R&D Systems, Minneapolis, MN). Samples were analyzed with a 4-laser Aurora Cytek (Cytek Biosciences, Fermont, CA) containing 16 violet, 14 blue, 10 yellow–green, and eight red channels (4L-16V-14B-10YG-8R). Spectral unmixing was performed using single-stained reference controls and the SpectroFlo unmixing wizard (Cytek Biosciences). One million total events were acquired per sample. Data were analyzed using FlowJo version 10 (BD Biosciences, Ashland, OR).

### *In vivo* alpha toxin production

Wells of 96-well Nunc Maxisorp ELISA plates (Thermo Fisher Scientific) were coated with 50 µL of 1 µg/mL 7B8 anti-alpha toxin capture antibody (University of Washington at St. Louis, St. Louis, MO) (109). Plates were wrapped in parafilm, incubated overnight at 4°C, washed three times with PBS-T, blocked with 1% BSA PBS-T, and placed on a shaker at room temperature for 2 h. Mice were euthanized, and eyes were harvested at 12, 24, or 36 h post-infection and transferred into bead beater tubes containing 1-mm glass beads containing lysis buffer (150 mM NaCl, 1 mM EDTA, 1 mM EGTA, 1% Triton X 100, and 20 mM Tris HCl pH 7.5, supplemented with cOmplete EDTA-free Protease Inhibitor, in PBS). Eyes were homogenized as described above and then placed on ice for 30 min. After blocking, plates were washed three times, and 50 uL of the eye homogenates or standards (starting at 160 ng/mL, followed by 12, 2-fold dilutions; University of Washington at St. Louis) was placed into the wells, and plates were incubated for 2 h at room temperature. Plates were then washed three times, followed by a 2-h incubation with 50 µL of the primary anti-alpha toxin rabbit polyclonal antibody (diluted 1:2,500) on a shaker. Plates were washed three times, and then 50 µL of an anti-rabbit IgG, HRP-linked detection antibody (diluted 1:4,000, Cell Signaling Technology, Danvers, MA) was added to each well, and then plates were incubated on a shaker for 1 h. Plates were washed four times, 50 µL of TMB substrate (Thermo Fisher) was added to each well, and plates were developed in the dark for 15 min. Reactions were stopped with 2 N sulfuric acid. Plates were read using the CLARIOStar plate reader (BMG Labtech, Cary, NC) at 450 nm. Due to minimal separations in the optical densities at concentrations < 0.625 ng/mL, 0.625 ng/mL was defined as the functional limit of detection for this assay.

### Statistics

The Kruskal-Wallis test with Dunn’s multiple correction test was used for statistical comparisons unless otherwise noted. Data were analyzed using GraphPad Prism 10 (GraphPad Software Inc., La Jolla CA). *P* values < 0.05 were considered significant. Each N value is a single biological replicate.

## References

[1] Graham JE, Moore JE, Jiru X, Moore JE, Goodall EA, Dooley JS, Hayes VE, Dartt DA, Downes CS, Moore TC. 2007. Ocular pathogen or commensal: a PCR-based study of surface bacterial flora in normal and dry eyes. Invest Ophthalmol Vis Sci 48:5616–5623. doi:10.1167/iovs.07-0588 18055811

[2] Astley RA, Mursalin MH, Coburn PS, Livingston ET, Nightengale JW, Bagaruka E, Hunt JJ, Callegan MC. 2023. Ocular bacterial infections: a ten-year survey and review of causative organisms based on the Oklahoma experience. Microorganisms 11:1802. doi:10.3390/microorganisms11071802 37512974 PMC10386592

[3] Durand ML. 2017. Bacterial and fungal endophthalmitis. Clin Microbiol Rev 30:597–613. doi:10.1128/CMR.00113-16 28356323 PMC5475221

[4] Astley RA, Coburn PS, Parkunan SM, Callegan MC. 2016. Modeling intraocular bacterial infections. Prog Retin Eye Res 54:30–48. doi:10.1016/j.preteyeres.2016.04.007 27154427 PMC4992594

[5] Callegan MC, Engelbert M, Parke DW II, Jett BD, Gilmore MS. 2002. Bacterial endophthalmitis: epidemiology, therapeutics, and bacterium-host interactions. Clin Microbiol Rev 15:111–124. doi:10.1128/CMR.15.1.111-124.2002 11781270 PMC118063

[6] Miller FC, Coburn PS, Huzzatul MM, LaGrow AL, Livingston E, Callegan MC. 2019. Targets of immunomodulation in bacterial endophthalmitis. Prog Retin Eye Res 73:100763. doi:10.1016/j.preteyeres.2019.05.004 31150824 PMC6881541

[7] Callegan MC, Gilmore MS, Gregory M, Ramadan RT, Wiskur BJ, Moyer AL, Hunt JJ, Novosad BD. 2007. Bacterial endophthalmitis: therapeutic challenges and host–pathogen interactions. Prog Retin Eye Res 26:189–203. doi:10.1016/j.preteyeres.2006.12.001 17236804 PMC1941835

[8] Chen KJ, Chen TL, Lai CC, Sun MH. 2008. Endophthalmitis: antibacterial activity of precipitates of vancomycin and ceftazidime. J Clin Microbiol 46:2149–2149. doi:10.1128/JCM.00728-08 18539703 PMC2446856

[9] Durand ML. 2013. Endophthalmitis. Clin Microbiol Infect 19:227–234. doi:10.1111/1469-0691.12118 23438028 PMC3638360

[10] Parkunan SM, Callegan MC. 2016. The pathogenesis of bacterial endophthalmitis, p 17–47. *In* Durand ML, Miller JW, Young LH (ed), Endophthalmitis. Springer International Publishing, Cham, Switzerland. doi:10.1007/978-3-319-29231-1_2.

[11] Huz JI, Mukkamala K, Pagan IR, Ritterband D, Shah M, Gentile RC, Engelbert M. 2017. Clinical outcomes and antibiotic susceptibilities of Staphylococcus aureus endophthalmitis. Graefes Arch Clin Exp Ophthalmol 255:651–656. doi:10.1007/s00417-016-3504-x 27757526

[12] Shirodkar AR, Flynn HW, Alliman K, Lalwani GA, Alabiad C, Moshfeghi AA, Miller D. 2010. The comparison of clinical outcomes of endophthalmitis from fluoroquinolone-resistant and susceptible bacteria. Clin Ophthalmol 4:211. doi:10.2147/OPTH.S9839 20463786 PMC2861925

[13] Major JC Jr, Engelbert M, Flynn HW Jr, Miller D, Smiddy WE, Davis JL. 2010. Staphylococcus aureus endophthalmitis: antibiotic susceptibilities, methicillin resistance, and clinical outcomes. Am J Ophthalmol 149:278–283. doi:10.1016/j.ajo.2009.08.023 19926069

[14] Schwartz SG, Flynn HW Jr. 2014. Update on the prevention and treatment of endophthalmitis. Expert Rev Ophthalmol 9:425–430. doi:10.1586/17469899.2014.951331 26609317 PMC4655603

[15] Novick RP, Geisinger E. 2008. Quorum sensing in Staphylococci. Annu Rev Genet 42:541–564. doi:10.1146/annurev.genet.42.110807.091640 18713030

[16] Booth MC, Atkuri RV, Nanda SK, Iandolo JJ, Gilmore MS. 1995. Accessory gene regulator controls Staphylococcus aureus virulence in endophthalmitis. Invest Ophthalmol Vis Sci 36:1828–1836. 7635657

[17] Booth MC, Cheung AL, Hatter KL, Jett BD, Callegan MC, Gilmore MS. 1997. Staphylococcal accessory regulator (sar) in conjunction with agr contributes to Staphylococcus aureus virulence in endophthalmitis. Infect Immun 65:1550–1556. doi:10.1128/iai.65.4.1550-1556.1997 9119503 PMC175169

[18] Abdelnour A, Arvidson S, Bremell T, Rydén C, Tarkowski A. 1993. The accessory gene regulator (agr) controls Staphylococcus aureus virulence in a murine arthritis model. Infect Immun 61:3879–3885. doi:10.1128/iai.61.9.3879-3885.1993 8359909 PMC281089

[19] Bronner S, Stoessel P, Gravet A, Monteil H, Prévost G. 2000. Variable expressions of Staphylococcus aureus bicomponent leucotoxins semiquantified by competitive reverse transcription-PCR. Appl Environ Microbiol 66:3931–3938. doi:10.1128/AEM.66.9.3931-3938.2000 10966411 PMC92241

[20] Tam K, Torres VJ. 2019. Staphylococcus aureus secreted toxins and extracellular enzymes. Microbiol Spectr 7:7. doi:10.1128/microbiolspec.gpp3-0039-2018 PMC6422052 30873936

[21] Berube BJ, Bubeck Wardenburg J. 2013. Staphylococcus aureus α-toxin: nearly a century of intrigue. Toxins (Basel) 5:1140–1166. doi:10.3390/toxins5061140 23888516 PMC3717774

[22] Wilke GA, Bubeck Wardenburg J. 2010. Role of a disintegrin and metalloprotease 10 in Staphylococcus aureus alpha-hemolysin-mediated cellular injury. Proc Natl Acad Sci USA 107:13473–13478. doi:10.1073/pnas.1001815107 20624979 PMC2922128

[23] Lubkin A, Lee WL, Alonzo F 3rd, Wang C, Aligo J, Keller M, Girgis NM, Reyes-Robles T, Chan R, O’Malley A, et al.. 2019. Staphylococcus aureus leukocidins target endothelial DARC to cause lethality in mice. Cell Host Microbe 25:463–470. doi:10.1016/j.chom.2019.01.015 30799265 PMC6468323

[24] Kumar A, Kumar A. 2015. Role of Staphylococcus aureus virulence factors in inducing inflammation and vascular permeability in a mouse model of bacterial endophthalmitis. PLoS One 10:e0128423. doi:10.1371/journal.pone.0128423 26053426 PMC4459968

[25] Siqueira J, Speeg-Schatz C, Freitas F, Sahel J, Monteil H, Prevost G. 1997. Channel-forming leucotoxins from Staphylococcus aureus cause severe inflammatory reactions in a rabbit eye model. J Med Microbiol 46:486–494. doi:10.1099/00222615-46-6-486 9350201

[26] Liu X, Heitz P, Roux M, Keller D, Bourcier T, Sauer A, Prévost G, Gaucher D. 2018. Panton–valentine leukocidin colocalizes with retinal ganglion and amacrine cells and activates glial reactions and microglial apoptosis. Sci Rep 8:2953. doi:10.1038/s41598-018-20590-z 29440661 PMC5811455

[27] Liu X, Roux MJ, Picaud S, Keller D, Sauer A, Heitz P, Prévost G, Gaucher D. 2018. Panton-valentine leucocidin proves direct neuronal targeting and its early neuronal and glial impacts a rabbit retinal explant model. Toxins (Basel) 10:455. doi:10.3390/toxins10110455 30400375 PMC6266138

[28] Wurtz M, Ruhland E, Liu X, Namer I-J, Mazzoleni V, Lipsker D, Keller D, Prévost G, Gaucher D. 2021. Panton-Valentine leucocidin of Staphylococcus aureus induces oxidative stress and neurotransmitter imbalance in a retinal explant model. Invest Ophthalmol Vis Sci 62:4. doi:10.1167/iovs.62.1.4 PMC7794257 33393970

[29] Supersac G, Piémont Y, Kubina M, Prévost G, Foster T. 1998. Assessment of the role of gamma-toxin in experimental endophthalmitis using a hlg-deficient mutant of Staphylococcus aureus. Microb Pathog 24:241–251. doi:10.1006/mpat.1997.0192 9533895

[30] Longoria-Gonzalez L, Coburn PS, Astley R, Chen Y, Callegan MC. 2025. Contribution of leukocidin ED to the pathogenesis of Staphylococcus aureus endophthalmitis. Invest Ophthalmol Vis Sci 66:11. doi:10.1167/iovs.66.5.11 PMC12060071 40323270

[31] Blake KJ, Baral P, Voisin T, Lubkin A, Pinho-Ribeiro FA, Adams KL, Roberson DP, Ma YC, Otto M, Woolf CJ, Torres VJ, Chiu IM. 2018. Staphylococcus aureus produces pain through pore-forming toxins and neuronal TRPV1 that is silenced by QX-314. Nat Commun 9:37. doi:10.1038/s41467-017-02448-6 29295977 PMC5750211

[32] D’Orazio TJ, Niederkorn JY. 1998. A novel role for TGF-beta and IL-10 in the induction of immune privilege. J Immunol 160:2089–2098. doi:10.4049/jimmunol.160.5.2089 9498745

[33] Talreja D, Singh PK, Kumar A. 2015. In vivo role of TLR2 and MyD88 signaling in eliciting innate immune responses in Staphylococcal endophthalmitis. Invest Ophthalmol Vis Sci 56:1719–1732. doi:10.1167/iovs.14-16087 25678692 PMC4356198

[34] Asbell PA, Sanfilippo CM, Mah FS. 2022. Antibiotic susceptibility of bacterial pathogens isolated from the aqueous and vitreous humour in the Antibiotic Resistance Monitoring in Ocular micRoorganisms (ARMOR) surveillance study: 2009-2020 update. J Glob Antimicrob Resist 29:236–240. doi:10.1016/j.jgar.2022.03.010 35339737

[35] Asbell PA, Sanfilippo CM, Sahm DF, DeCory HH. 2020. Trends in antibiotic resistance among ocular microorganisms in the United States from 2009 to 2018. JAMA Ophthalmol 138:439–450. doi:10.1001/jamaophthalmol.2020.0155 32271355 PMC7146550

[36] Zaidi T, Zaidi T, Yoong P, Pier GB. 2013. Staphylococcus aureus corneal infections: effect of the Panton-Valentine leukocidin (PVL) and antibody to PVL on virulence and pathology. Invest Ophthalmol Vis Sci 54:4430–4438. doi:10.1167/iovs.13-11701 23737477 PMC3700385

[37] Dajcs JJ, Austin MS, Sloop GD, Moreau JM, Hume EB, Thompson HW, McAleese FM, Foster TJ, O’Callaghan RJ. 2002. Corneal pathogenesis of Staphylococcus aureus strain Newman. Invest Ophthalmol Vis Sci 43:1109–1115. 11923253

[38] Callegan MC, Engel LS, Hill JM, O’Callaghan RJ. 1994. Corneal virulence of Staphylococcus aureus: roles of alpha-toxin and protein A in pathogenesis. Infect Immun 62:2478–2482. doi:10.1128/iai.62.6.2478-2482.1994 8188373 PMC186534

[39] Spaan AN, Henry T, van Rooijen WJM, Perret M, Badiou C, Aerts PC, Kemmink J, de Haas CJC, van Kessel KPM, Vandenesch F, Lina G, van Strijp JAG. 2013. The staphylococcal toxin panton-valentine leukocidin targets human C5a receptors. Cell Host Microbe 13:584–594. doi:10.1016/j.chom.2013.04.006 23684309

[40] DuMont AL, Yoong P, Day CJ, Alonzo F III, McDonald WH, Jennings MP, Torres VJ. 2013. Staphylococcus aureus LukAB cytotoxin kills human neutrophils by targeting the CD11b subunit of the integrin Mac-1. Proc Natl Acad Sci USA 110:10794–10799. doi:10.1073/pnas.1305121110 23754403 PMC3696772

[41] Malachowa N, Whitney AR, Kobayashi SD, Sturdevant DE, Kennedy AD, Braughton KR, Shabb DW, Diep BA, Chambers HF, Otto M, DeLeo FR. 2011. Global changes in Staphylococcus aureus gene expression in human blood. PLoS One 6:e18617. doi:10.1371/journal.pone.0018617 21525981 PMC3078114

[42] DuMont AL, Yoong P, Surewaard BGJ, Benson MA, Nijland R, van Strijp JAG, Torres VJ. 2013. Staphylococcus aureus elaborates leukocidin AB to mediate escape from within human neutrophils. Infect Immun 81:1830–1841. doi:10.1128/IAI.00095-13 23509138 PMC3648020

[43] Alonzo III F, Benson MA, Chen J, Novick RP, Shopsin B, Torres VJ. 2012. Staphylococcus aureus leucocidin ED contributes to systemic infection by targeting neutrophils and promoting bacterial growth in vivo. Mol Microbiol 83:423–435. doi:10.1111/j.1365-2958.2011.07942.x 22142035 PMC3258504

[44] Bishop PN. 2000. Structural macromolecules and supramolecular organisation of the vitreous gel. Prog Retin Eye Res 19:323–344. doi:10.1016/s1350-9462(99)00016-6 10749380

[45] Lundquist O, Osterlin S. 1994. Glucose concentration in the vitreous of nondiabetic and diabetic human eyes. Graefes Arch Clin Exp Ophthalmol 232:71–74. doi:10.1007/BF00171666 8157178

[46] Karwoski CJ, Xu X. 1999. Current source-density analysis of light-evoked field potentials in rabbit retina. Vis Neurosci 16:369–377. doi:10.1017/s0952523899162163 10367970

[47] Cheng L, Bu H, Portillo J-A, Li Y, Subauste CS, Huang SS, Kern TS, Lin F. 2013. Modulation of retinal müller cells by complement receptor C5aR. Invest Ophthalmol Vis Sci 54:8191. doi:10.1167/iovs.13-12428 24265019 PMC3867184

[48] Spaan AN, van Strijp JA, Torres VJ. 2017. Leukocidins: staphylococcal bi-component pore-forming toxins find their receptors. Nat Rev Microbiol 15:435–447. doi:10.1038/nrmicro.2017.27 28420883 PMC5621924

[49] Girgis DO, Sloop GD, Reed JM, O’Callaghan RJ. 2005. Effects of toxin production in a murine model of Staphylococcus aureus keratitis. Invest Ophthalmol Vis Sci 46:2064. doi:10.1167/iovs.04-0897 15914624

[50] O’Callaghan RJ, Callegan MC, Moreau JM, Green LC, Foster TJ, Hartford OM, Engel LS, Hill JM. 1997. Specific roles of alpha-toxin and beta-toxin during Staphylococcus aureus corneal infection. Infect Immun 65:1571–1578. doi:10.1128/iai.65.5.1571-1578.1997 9125532 PMC175175

[51] Kielian T, Cheung A, Hickey WF. 2001. Diminished virulence of an alpha-toxin mutant of Staphylococcus aureus in experimental brain abscesses. Infect Immun 69:6902–6911. doi:10.1128/IAI.69.11.6902-6911.2001 11598065 PMC100070

[52] Brady RA, Mocca CP, Prabhakara R, Plaut RD, Shirtliff ME, Merkel TJ, Burns DL. 2013. Evaluation of genetically inactivated alpha toxin for protection in multiple mouse models of Staphylococcus aureus Infection. PLoS One 8:e63040. doi:10.1371/journal.pone.0063040 23658662 PMC3639205

[53] Thurlow LR, Stephens AC, Hurley KE, Richardson AR. 2020. Lack of nutritional immunity in diabetic skin infections promotes Staphylococcus aureus virulence. Sci Adv 6:eabc5569. doi:10.1126/sciadv.abc5569 33188027 PMC7673755

[54] Bartlett AH, Foster TJ, Hayashida A, Park PW. 2008. α‐toxin facilitates the generation of CXC chemokine gradients and stimulates neutrophil homing in Staphylococcus aureus pneumonia . J Infect Dis 198:1529–1535. doi:10.1086/592758 18823272 PMC12822823

[55] Cho JS, Guo Y, Ramos RI, Hebroni F, Plaisier SB, Xuan C, Granick JL, Matsushima H, Takashima A, Iwakura Y, Cheung AL, Cheng G, Lee DJ, Simon SI, Miller LS. 2012. Neutrophil-derived IL-1β Is sufficient for abscess formation in immunity against Staphylococcus aureus in mice. PLoS Pathog 8:e1003047. doi:10.1371/journal.ppat.1003047 23209417 PMC3510260

[56] Kielian T, Bearden ED, Baldwin AC, Esen N. 2004. IL-1 and TNF-alpha play a pivotal role in the host immune response in a mouse model of Staphylococcus aureus-induced experimental brain abscess. J Neuropathol Exp Neurol 63:381–396. doi:10.1093/jnen/63.4.381 15099027

[57] Youn C, Pontaza C, Wang Y, Dikeman DA, Joyce DP, Alphonse MP, Wu MJ, Nolan SJ, Anany MA, Ahmadi M, Young J, Tocaj A, Garza LA, Wajant H, Miller LS, Archer NK. 2023. Neutrophil-intrinsic TNF receptor signaling orchestrates host defense against Staphylococcus aureus. Sci Adv 9:eadf8748. doi:10.1126/sciadv.adf8748 37327341 PMC10275602

[58] Kumar A, Singh PK, Ahmed Z, Singh S, Kumar A. 2022. Essential role of NLRP3 Inflammasome in mediating IL-1β production and the pathobiology of Staphylococcus aureus endophthalmitis. Infect Immun 90:e0010322. doi:10.1128/iai.00103-22 35404106 PMC9119078

[59] Holzinger D, Gieldon L, Mysore V, Nippe N, Taxman DJ, Duncan JA, Broglie PM, Marketon K, Austermann J, Vogl T, Foell D, Niemann S, Peters G, Roth J, Löffler B. 2012. Staphylococcus aureus Panton-Valentine leukocidin induces an inflammatory response in human phagocytes via the NLRP3 inflammasome. J Leukoc Biol 92:1069–1081. doi:10.1189/jlb.0112014 22892107 PMC3476237

[60] Melehani JH, James DBA, DuMont AL, Torres VJ, Duncan JA. 2015. Staphylococcus aureus leukocidin A/B (LukAB) kills human monocytes via host NLRP3 and ASC when extracellular, but not intracellular. PLoS Pathog 11:e1004970. doi:10.1371/journal.ppat.1004970 26069969 PMC4466499

[61] Muñoz-Planillo R, Franchi L, Miller LS, Núñez G. 2009. A critical role for hemolysins and bacterial lipoproteins in Staphylococcus aureus-induced activation of the Nlrp3 inflammasome. J Immunol 183:3942–3948. doi:10.4049/jimmunol.0900729 19717510 PMC2762867

[62] Craven RR, Gao X, Allen IC, Gris D, Bubeck Wardenburg J, McElvania-Tekippe E, Ting JP, Duncan JA. 2009. Staphylococcus aureus alpha-hemolysin activates the NLRP3-inflammasome in human and mouse monocytic cells. PLoS One 4:e7446. doi:10.1371/journal.pone.0007446 19826485 PMC2758589

[63] Liboro K, Chau JT, Begando JD, Abbondante S, Lackner A, Sun Y, Marshall ME, Ly N, Johnson VD, Dubyak GR, Gilmore M, McNulty R, Andre C, Pearlman E. 2025. α-hemolysin polymorphisms in methicillin-resistant Staphylococcus aureus clinical isolates regulate ADAM10-dependent neutrophil IL-1β secretion. bioRxiv:2025.12.13.694139. doi:10.64898/2025.12.13.694139

[64] Ramadan RT, Moyer AL, Callegan MC. 2008. A role for tumor necrosis factor-alpha in experimental Bacillus cereus endophthalmitis pathogenesis. Invest Ophthalmol Vis Sci 49:4482–4489. doi:10.1167/iovs.08-2085 18586878 PMC2574773

[65] Hume EB, Cole N, Garthwaite LL, Khan S, Willcox MD. 2006. A protective role for IL-6 in staphylococcal microbial keratitis. Invest Ophthalmol Vis Sci 47:4926–4930. doi:10.1167/iovs.06-0340 17065508

[66] Brubaker AL, Kovacs EJ. 2013. G-CSF enhances resolution of Staphylococcus aureus wound infection in an age-dependent manner. Shock 40:327–333. doi:10.1097/SHK.0b013e3182a43651 23856924 PMC3792575

[67] Martin KR, Wong HL, Witko-Sarsat V, Wicks IP. 2021. G-CSF - a double edge sword in neutrophil mediated immunity. Semin Immunol 54:101516. doi:10.1016/j.smim.2021.101516 34728120

[68] Coburn PS, Parrott AC, Miller FC, LaGrow AL, Mursalin MH, Callegan MC. 2023. The role of C-X-C chemokines in Staphylococcus aureus endophthalmitis. Invest Ophthalmol Vis Sci 64:10. doi:10.1167/iovs.64.3.10 PMC9988700 36867134

[69] Onogawa T. 2002. Staphylococcal alpha-toxin synergistically enhances inflammation caused by bacterial components. FEMS Immunol Med Microbiol 33:15–21. doi:10.1111/j.1574-695X.2002.tb00566.x 11985963

[70] Fuhr A, Goerlich M, Biedritzky A, Kleinmaier C, Heck-Swain KL, Gamper-Tsigaras J, Ngamsri KC, Konrad F, Koeppen M. 2025. Neutrophil ADAM10 promotes migration and inflammation in ARDS by modulating adhesion and chemokine signaling. Mucosal Immunol 18:1353–1365. doi:10.1016/j.mucimm.2025.09.003 40947020

[71] Nygaard TK, Pallister KB, Zurek OW, Voyich JM. 2013. The impact of α-toxin on host cell plasma membrane permeability and cytokine expression during human blood infection by CA-MRSA USA300. J Leukoc Biol 94:971–979. doi:10.1189/jlb.0213080 24026286 PMC3800068

[72] Didangelos A. 2020. COVID-19 hyperinflammation: what about neutrophils? mSphere 5:e00367-20. doi:10.1128/mSphere.00367-20 32581077 PMC7316488

[73] Valeva A, Walev I, Pinkernell M, Walker B, Bayley H, Palmer M, Bhakdi S. 1997. Transmembrane beta-barrel of staphylococcal alpha-toxin forms in sensitive but not in resistant cells. Proc Natl Acad Sci USA 94:11607–11611. doi:10.1073/pnas.94.21.11607 9326657 PMC23553

[74] Becker RE, Berube BJ, Sampedro GR, DeDent AC, Bubeck Wardenburg J. 2014. Tissue-specific patterning of host innate immune responses by Staphylococcus aureus α-toxin. J Innate Immun 6:619–631. doi:10.1159/000360006 24820433 PMC4464663

[75] Yang F, Suo M, Weli H, Wong M, Junidi A, Cummings C, Johnson R, Mallory K, Liu AY, Greenberg ZJ, Schuettpelz LG, Miller MJ, Luke CJ, Randolph GJ, Zinselmeyer BH, Wardenburg JB, Clemens RA. 2023. Staphylococcus aureus α-toxin impairs early neutrophil localization via electrogenic disruption of store-operated calcium entry. Cell Rep 42:113394. doi:10.1016/j.celrep.2023.113394 37950870 PMC10731421

[76] Powers ME, Becker REN, Sailer A, Turner JR, Bubeck Wardenburg J. 2015. Synergistic action of Staphylococcus aureus α-toxin on platelets and myeloid lineage cells contributes to lethal sepsis. Cell Host Microbe 17:775–787. doi:10.1016/j.chom.2015.05.011 26067604 PMC4642999

[77] Yan X, Lin J, Rolfs A, Luo J. 2011. Differential expression of the ADAMs in developing chicken retina. Dev Growth Differ 53:726–739. doi:10.1111/j.1440-169X.2011.01282.x 21671920

[78] Toonen JA, Ronchetti A, Sidjanin DJ. 2016. A disintegrin and metalloproteinase10 (ADAM10) regulates NOTCH signaling during early retinal development. PLoS One 11:e0156184. doi:10.1371/journal.pone.0156184 27224017 PMC4880208

[79] Cisneros E, di Marco F, Rueda-Carrasco J, Lillo C, Pereyra G, Martín-Bermejo MJ, Vargas A, Sanchez R, Sandonís Á, Esteve P, Bovolenta P. 2020. Sfrp1 deficiency makes retinal photoreceptors prone to degeneration. Sci Rep 10:5115. doi:10.1038/s41598-020-61970-8 32198470 PMC7083943

[80] Alli-Shaik A, Qiu B, Lai SL, Cheung N, Tan G, Neo SP, Tan A, Cheung CMG, Hong W, Wong TY, Wang X, Gunaratne J. 2022. System-wide vitreous proteome dissection reveals impaired sheddase activity in diabetic retinopathy. Theranostics 12:6682–6704. doi:10.7150/thno.72947 36185601 PMC9516227

[81] Bisen S, Gogoi P, Sharma A, Mukhopadhyay CS, Singh NK. 2025. A disintegrin and metalloproteinase 10 regulates ephrin B2-mediated endothelial cell sprouting and ischemic retinopathy. Am J Pathol 195:1311–1327. doi:10.1016/j.ajpath.2025.03.007 40252970 PMC12264544

[82] Kim D, Ko HS, Park GB, Hur DY, Kim YS, Yang JW. 2017. Vandetanib and ADAM inhibitors synergistically attenuate the pathological migration of EBV-infected retinal pigment epithelial cells by regulating the VEGF-mediated MAPK pathway. Exp Ther Med 13:1415–1425. doi:10.3892/etm.2017.4110 28413487 PMC5377331

[83] Hume EB, Dajcs JJ, Moreau JM, O’Callaghan RJ. 2000. Immunization with alpha-toxin toxoid protects the cornea against tissue damage during experimental Staphylococcus aureus keratitis. Infect Immun 68:6052–6055. doi:10.1128/IAI.68.10.6052-6055.2000 10992521 PMC101573

[84] Caballero AR, Foletti DL, Bierdeman MA, Tang A, Arana AM, Hasa-Moreno A, Sangalang ERB, O’Callaghan RJ. 2015. Effectiveness of alpha-toxin fab monoclonal antibody therapy in limiting the pathology of Staphylococcus aureus keratitis. Ocul Immunol Inflamm 23:297–303. doi:10.3109/09273948.2014.920035 24912088

[85] Coburn PS, Miller FC, LaGrow AL, Land C, Mursalin H, Livingston E, Amayem O, Chen Y, Gao W, Zhang L, Callegan MC. 2019. Disarming pore-forming toxins with biomimetic nanosponges in intraocular infections. mSphere 4:e00262-19. doi:10.1128/mSphere.00262-19 31092603 PMC6520441

[86] Hu CM, Fang RH, Copp J, Luk BT, Zhang L. 2013. A biomimetic nanosponge that absorbs pore-forming toxins. Nature Nanotech 8:336–340. doi:10.1038/nnano.2013.54 PMC3648601 23584215

[87] Reyes-Robles T, Alonzo F III, Kozhaya L, Lacy DB, Unutmaz D, Torres VJ. 2013. Staphylococcus aureus leukotoxin ed targets the chemokine receptors CXCR1 and CXCR2 to kill leukocytes and promote infection. Cell Host & Microbe 14:453–459. doi:10.1016/j.chom.2013.09.005 24139401 PMC3876884

[88] Spaan AN, Vrieling M, Wallet P, Badiou C, Reyes-Robles T, Ohneck EA, Benito Y, de Haas CJC, Day CJ, Jennings MP, Lina G, Vandenesch F, van Kessel KPM, Torres VJ, van Strijp JAG, Henry T. 2014. The staphylococcal toxins γ-haemolysin AB and CB differentially target phagocytes by employing specific chemokine receptors. Nat Commun 5:5438. doi:10.1038/ncomms6438 25384670 PMC4228697

[89] Graves SF, Kobayashi SD, Braughton KR, Whitney AR, Sturdevant DE, Rasmussen DL, Kirpotina LN, Quinn MT, DeLeo FR. 2012. Sublytic concentrations of Staphylococcus aureus panton-valentine leukocidin alter human PMN gene expression and enhance bactericidal capacity. J Leukoc Biol 92:361–374. doi:10.1189/jlb.1111575 22581932 PMC3395418

[90] Perret M, Badiou C, Lina G, Burbaud S, Benito Y, Bes M, Cottin V, Couzon F, Juruj C, Dauwalder O, Goutagny N, Diep BA, Vandenesch F, Henry T. 2012. Cross-talk between Staphylococcus aureus leukocidins-intoxicated macrophages and lung epithelial cells triggers chemokine secretion in an inflammasome-dependent manner. Cell Microbiol 14:1019–1036. doi:10.1111/j.1462-5822.2012.01772.x 22329718

[91] Yoong P, Torres VJ. 2015. Counter inhibition between leukotoxins attenuates Staphylococcus aureus virulence. Nat Commun 6:8125. doi:10.1038/ncomms9125 26330208 PMC4562310

[92] Nilsson IM, Hartford O, Foster T, Tarkowski A. 1999. Alpha-toxin and gamma-toxin jointly promote Staphylococcus aureus virulence in murine septic arthritis. Infect Immun 67:1045–1049. doi:10.1128/IAI.67.3.1045-1049.1999 10024541 PMC96427

[93] Scherr TD, Hanke ML, Huang O, James DB, Horswill AR, Bayles KW, Fey PD, Torres VJ, Kielian T. 2015. Staphylococcus aureus biofilms induce macrophage dysfunction through leukocidin ab and alpha-toxin. mBio 6:e01021-15. doi:10.1128/mBio.01021-15 26307164 PMC4550693

[94] Afzal M, Vijay AK, Stapleton F, Willcox M. 2022 a. Virulence genes of Staphylococcus aureus associated with keratitis, conjunctivitis, and contact lens–associated inflammation . Trans Vis Sci Tech 11:5. doi:10.1167/tvst.11.7.5 PMC9279920 35802366

[95] Afzal M, Vijay AK, Stapleton F, Willcox MD. 2022. Genomics of Staphylococcus aureus strains isolated from infectious and non-infectious ocular conditions. Antibiotics (Basel) 11:1011. doi:10.3390/antibiotics11081011 36009880 PMC9405196

[96] Booth MC, Pence LM, Mahasreshti P, Callegan MC, Gilmore MS. 2001. Clonal associations among Staphylococcus aureus isolates from various sites of infection. Infect Immun 69:345–352. doi:10.1128/IAI.69.1.345-352.2001 11119523 PMC97889

[97] Vitko NP, Richardson AR. 2013. Laboratory maintenance of methicillin‐resistant Staphylococcus aureus (MRSA) . CP Microbiology 28:9C. doi:10.1002/9780471729259.mc09c02s28 PMC4070006 23408135

[98] Livingston ET, Mursalin MH, Coburn PS, Astley R, Miller FC, Amayem O, Lereclus D, Callegan MC. 2021. Immune inhibitor a metalloproteases contribute to virulence in Bacillus endophthalmitis. Infect Immun 89:e0020121. doi:10.1128/IAI.00201-21 34097460 PMC8447934

[99] Mursalin MH, Livingston E, Coburn PS, Miller FC, Astley R, Callegan MC. 2021. Intravitreal injection and quantitation of infection parameters in a mouse model of bacterial endophthalmitis. J Vis Exp 168. doi:10.3791/61749 PMC8107885 33616100

[100] Singh PK, Singh S, Wright RE, Rattan R, Kumar A. 2020. Aging, but not sex and genetic diversity, impacts the pathobiology of bacterial endophthalmitis. Invest Ophthalmol Vis Sci 61:5. doi:10.1167/iovs.61.14.5 PMC7718809 33263715

[101] Mursalin MH, Astley R, Coburn PS, Miller FC, Callegan MC. 2022. Roles of CCL2 and CCL3 in intraocular inflammation during Bacillus endophthalmitis. Exp Eye Res 224:109213. doi:10.1016/j.exer.2022.109213 36063964 PMC9826602

[102] Mursalin MH, Coburn PS, Miller FC, Livingston ET, Astley R, Callegan MC. 2021. C-X-C chemokines influence intraocular inflammation during Bacillus endophthalmitis. Invest Ophthalmol Vis Sci 62:14. doi:10.1167/iovs.62.14.14 PMC8606850 34784411

[103] Kamath MM, Lightfoot JD, Adams EM, Kiser RM, Wells BL, Fuller KK. 2023. The aspergillus fumigatus UPR is variably activated across nutrient and host environments and is critical for the establishment of corneal infection. PLoS Pathog 19:e1011435. doi:10.1371/journal.ppat.1011435 37906600 PMC10637725

[104] Callegan MC, Jett BD, Hancock LE, Gilmore MS. 1999. Role of hemolysin BL in the pathogenesis of extraintestinal Bacillus cereus infection assessed in an endophthalmitis model. Infect Immun 67:3357–3366. doi:10.1128/IAI.67.7.3357-3366.1999 10377113 PMC116518

[105] Mursalin MH, Coburn PS, Longoria-Gonzalez L, Astley R, Fischetti VA, Callegan MC. 2025. Novel anti-microbial/anti-inflammatory combination improves clinical outcome of Bacillus cereus endophthalmitis. Invest Ophthalmol Vis Sci 66:39. doi:10.1167/iovs.66.1.39 PMC11741065 39813055

[106] Mursalin MH, Astley R, Coburn PS, Bagaruka E, Hunt JJ, Fischetti VA, Callegan MC. 2023. Therapeutic potential of Bacillus phage lysin PlyB in ocular infections. mSphere 8:e0004423. doi:10.1128/msphere.00044-23 37273201 PMC10449515

[107] Parrott AC, Coburn PS, Miller FC, LaGrow AL, Mursalin MH, Callegan MC. 2024. The role of ccl chemokines in experimental Staphylococcus aureus endophthalmitis. Invest Ophthalmol Vis Sci 65:12. doi:10.1167/iovs.65.6.12 PMC11160947 38842829

[108] Bhatt Y, Hunt DM, Carvalho LS. 2023. The origins of the full-field flash electroretinogram b-wave. Front Mol Neurosci 16:1153934. doi:10.3389/fnmol.2023.1153934 37465364 PMC10351385

[109] Tomaszewski KL, Blanchard M, Olaniyi R, Brenton HR, Hayes S, Fatma F, Amarasinghe GK, Cho BK, Goo YA, DeDent AC, Fritz SA, Wardenburg JB. 2024. Enhanced Staphylococcus aureus protection by uncoupling of the α-toxin-ADAM10 interaction during murine neonatal vaccination. Nat Commun 15:8702. doi:10.1038/s41467-024-52714-7 39379345 PMC11461939

